# Guidelines on deep vein thrombosis of the Brazilian Society of Angiology and Vascular Surgery

**DOI:** 10.1590/1677-5449.202301072

**Published:** 2024-09-03

**Authors:** Marcone Lima Sobreira, Marcos Áreas Marques, Adilson Ferraz Paschoa, Alcides José Araújo Ribeiro, Ivan Benaduce Casella, Marcelo Calil Burihan, Marcelo Fernando Matielo, Rafael de Athayde Soares, Walter Junior Boin de Araujo, Edwaldo Edner Joviliano, Julio Cesar Peclat de Oliveira

**Affiliations:** 1 Universidade Estadual Paulista “Júlio de Mesquita Filho” – UNESP, Departamento de Cirurgia e Ortopedia, São Paulo, SP, Brasil.; 2 Universidade do Estado do Rio de Janeiro – UERJ, Departamento de Medicina Interna, Rio de Janeiro, RJ, Brasil.; 3 Associação Portuguesa de Beneficência de São Paulo, Centro de Cirurgia Vascular Integrada, Real e Benemérita, São Paulo, SP, Brasil.; 4 Hospital de Base do Distrito Federal, Serviço de Cirurgia Vascular, Brasília, DF, Brasil.; 5 Universidade de São Paulo – USP, Departamento de Cirurgia, São Paulo, SP, Brasil.; 6 Hospital Santa Marcelina, Departamento de Cirurgia Vascular, Endovascular e Ecografia Vascular, São Paulo, SP, Brasil.; 7 Hospital do Servidor Público Estadual de São Paulo, Departamento de Cirurgia Vascular e Endovascular, São Paulo, SP, Brasil.; 8 Universidade Federal do Paraná – UFPR, Hospital das Clínicas – HC, Curitiba, PR, Brasil.; 9 Sociedade Brasileira de Angiologia e de Cirurgia Vascular – SBACV-PR, Curitiba, PR, Brasil.; 10 Universidade de São Paulo – USP, Faculdade de Medicina de Ribeirão Preto – FMRP, Ribeirão Preto, SP, Brasil.; 11 Sociedade Brasileira de Angiologia e de Cirurgia Vascular – SBACV-SP, São Paulo, SP, Brasil.; 12 Universidade Federal do Estado do Rio de Janeiro – UNIRIO, Programa de Pós-graduação de Cirurgia Vascular, Rio de Janeiro, RJ, Brasil.

**Keywords:** deep vein thrombosis, venous thromboembolism, duplex Doppler ultrasonography, anticoagulants, fibrinolysis, vena cava filters

## Abstract

Deep vein thrombosis is one of the main causes of inpatient and outpatient morbidity, both in medical and surgical patients, significantly impacting mortality statistics and requiring prompt diagnosis so that treatment can be initiated immediately. This document was prepared and reviewed by 11 specialists certified by the Brazilian Society of Angiology and Vascular Surgery, who searched the main databases for the best evidence on the diagnostic (physical examination, imaging) and therapeutic approaches (heparin, coumarins, direct oral anticoagulants, fibrinolytics) to the disease.

## INTRODUCTION

Deep vein thrombosis (DVT) is characterized by thrombus formation within the deep veins, with partial obstruction or occlusion, and is more common in the lower extremities – in 80-95% of cases.^1,2^ The main complications resulting from this disease are chronic venous insufficiency/post-thrombotic syndrome (PTS) (edema and/or pain in the lower extremities, pigmentation changes, skin ulceration) and pulmonary embolism (PE). The latter is of high clinical importance, as it has a high mortality rate.^1,2^

Approximately 5% to 15% of individuals with untreated DVT may die of PE.^3^ DVT or PE may occur in 2 per 1000 person-years, with a recurrence rate of up to 25%.^3^ Prompt initiation of diagnostic investigations and treatment is crucial to avoid these complications.

## RISK FACTORS

The main factors directly associated with the genesis of thrombi are blood stasis, endothelial injury, and hypercoagulable states.^2^ Therefore, risk factors for DVT include advanced age, cancer, surgical procedures, immobilization, estrogen use, pregnancy-puerperal cycle, and inherited or acquired hypercoagulable states.^3,4^ Its incidence increases proportionally with age, suggesting that this is the most important determinant of a first thrombotic event.^5^

For didactic purposes, risk factors can be classified as follows:^6^

- Inherited/idiopathic: activated protein C resistance (mainly factor V Leiden); prothrombin gene *G20210A* mutation; antithrombin deficiency; protein C deficiency; protein S deficiency; elevated factor VIII levels; and increased fibrinogen levels;
- Acquired/provoked: antiphospholipid syndrome (APS); cancer; paroxysmal nocturnal hemoglobinuria; age > 65 years; overweight and obesity; pregnancy and puerperium; myeloproliferative diseases (eg, polycythemia vera and essential thrombocythemia); nephrotic syndrome; hyperviscosity (eg, in Waldenström macroglobulinemia and multiple myeloma); Behçet’s disease; trauma; surgery; immobilization; and estrogen therapy.

## CLASSIFICATION

Lower extremity DVT is divided according to its location into:

- Proximal: when it involves the iliac and/or femoral and/or popliteal veins;
- Distal: when it involves the veins located below the popliteal vein.

The risk of PE and magnitude of PTS are greater when resulting from proximal DVT. However, there is an up to 20% risk of progression of distal DVT to proximal segments, which makes its diagnosis and treatment similar to that of proximal DVT.^7^ Therefore, classifying the type of suspected DVT is important to guide treatment strategies.

## DIAGNOSIS

### Physical examination

When present, the main signs and symptoms are pain, edema, erythema, cyanosis, dilated superficial veins, elevated body temperature, and muscle cramps.^7^ The main factors related to the development of DVT, associated with pain and edema, can be grouped into clinical prediction models.^8^

No clinical assessment alone is sufficient to diagnose or rule out DVT,^1^ as clinical findings are related to the disease in only 50% of cases.^2^ Existing literature recommends history taking and physical examination, combined with laboratory tests and imaging.^1,8,9^ The best studied clinical DVT prediction system is the Wells score (WS).^9,10^

### Wells score (WS)

WS is a clinical prediction model based on signs and symptoms, risk factors, and alternative diagnoses that estimates the pretest probability of DVT (Table 1). This score has proven useful in the initial management of patients with suspected DVT.^8,9,12^ In its first version, WS categorizes patients into low, moderate, and high probability of DVT with a prevalence of 5% (95% CI, 4-8%), 17% (95% CI, 13-23%), and 53% (95% CI, 44-61%), respectively.^10^

**Table 1:** Thrombolytic therapy plus anticoagulation compared with anticoagulation alone in patients with extensive proximal DVT.

Outcome	Relative risk (95% CI)	Risk with anticoagulation	Risk difference with thrombolytic therapy + anticoagulation
Mortality	0.77 (0.26-2.28)	9 per 1000	2 fewer per 1000
Post-thrombotic syndrome	0.71 (0.60-0.085)	641 per 1000	186 fewer per 1000
Major bleeding	1.85 (1.45-2.44)	36 per 1000	31 more per 1000

DVT = deep vein thrombosis. Adapted from the American Society of Hematology 2020 guidelines.^11^

WS should be used in combination with additional diagnostic methods, such as color Doppler ultrasound (CDU) with compression of the entire lower-extremity venous system (patients with a high probability WS), and measurement of D-dimer (DD) (patients with a low probability WS).^1,9^ A negative CDU followed by a negative DD test result allows us to safely rule out DVT.^8^ Geersing et al.,^8^ in a meta-analysis, defined that, in patients with a WS ≤ 1 and a negative DD test result, the probability of DVT is less than 2% and it can be safely excluded in different groups of patients, except for those with cancer and recurrent DVT. WS produces better results in the assessment of young patients without comorbidities or previous history of venous thromboembolism (VTE) than in other patients.^13^ For recurrent DVT, it is recommended to use the modified WS (adding one extra point to patients with prior history of DVT).^8^

### Laboratory testing

#### DD in the diagnosis and monitoring of VTE

DD is a molecular marker that results from the dissolution of fibrin. It is usually elevated in thrombotic events, but it can also be elevated in other situations such as pregnancy, cancer, trauma, infection, and other pro-inflammatory processes.^1^ The sensitivity of this marker ranges from 94% to 100%, so the absence of elevated serum DD levels rules out a diagnosis of DVT in 97% to 98% of cases. DD levels are grouped into negative (< 350 ng/mL), intermediate (351-500 ng/mL), and positive (> 500 ng/mL).^13,14^

DD levels increase with increasing age, which reduces the specificity of the test. A systematic review published in 2014 demonstrated the need for an age-adjust DD (age multiplied by 10 mcg/L) in patients older than 50 years, and this increased specificity without changing the sensitivity of the laboratory test.^14^ DD levels also increase with pregnancy, which can reduce the specificity of the test if DD measurement is necessary in a possible diagnosis of DVT.^14,15^ In a small sample of asymptomatic women, none of the pregnant women had DD levels lower than 0.50 mg/L in the third trimester, and the authors concluded that higher thresholds should be used for the first (0.75 mg/L), second (1 mg/L), and third (1.5 mg/L) trimesters.^16^

### Recommendation 1

When combined with a negative DD test result, it is possible to rule out DVT in patients who have a low probability WS. Class I, level A^8^

#### DD and the risk of VTE

In critically ill hospitalized patients, elevated DD is associated with an increased risk of VTE, regardless of age, sex, race, body mass index, and clinical disease.^17^ The MAGELLAN trial evaluated 7581 critically ill hospitalized patients and observed that the incidence of VTE was 3.5 times higher in patients with serum DD levels greater than or equal to twice the upper limit of normal (odds ratio [OR] 2.29; 95% CI, 1.75-2.98).^17^ In the general population, elevated DD was associated with an increased risk of a first VTE episode in a study evaluating 923 patients during 8 years of follow-up.^18^

In patients with cancer, DD levels are considerably elevated in patients presenting with a first episode of VTE.^19^ In a study of 821 patients with cancer, a 2-fold increase in DD levels was associated with a 1.3-fold increase in the incidence of VTE both in univariate (95% CI, 1.1-1.5; p<0.001) and multivariate analysis (hazard ratio [HR] 1.3; 95% CI, 1.2-1.6; p<0.001).^20^ The association between elevated DD and the occurrence of a first VTE episode has been validated in several studies, including colorectal, lung, and gynecologic cancer.^21^

#### DD and VTE recurrence

Regarding VTE recurrence and DD levels, there is a representative systematic review, involving 1888 patients with a first episode of unprovoked VTE, which demonstrated that patients who maintained an elevated DD level after discontinuation of anticoagulant therapy had an annual rate of recurrent VTE of 8.9%, compared with 3.5% in patients with a normal DD level.^22^ A meta-analysis of the same population showed an annual rate of recurrent VTE of 8.8% in patients with an elevated DD level, compared with 3.7% in patients with a normal DD level.^23^ A recent study demonstrated an association between elevated DD levels and the risk of recurrent VTE from 3 weeks (HR 1.4; 1.06-1.84) up to 15 months (HR 1.26; 1.01-1. 57) after discontinuation of anticoagulant therapy.^24^ Although there are no international or national guidelines advising the maintenance or discontinuation of anticoagulants based on DD levels, it is recommended that DD levels be measured 1 month after discontinuing anticoagulant therapy. If the DD level remains elevated, it is necessary to consider the results of these 2 articles that demonstrate an almost 2-fold higher incidence of recurrent VTE in patients with elevated DD levels than in those with normal levels.

### Recommendation 2

Persistently elevated serum DD levels after stopping anticoagulation may indicate risk of recurrence, but they do not predict greater or lesser risk of recurrence of a thrombotic event. Class I, level A.^23^

#### DD and COVID-19

The discussion about DD and the risk of VTE has gained special attention due to the COVID-19 pandemic, as high DD levels were observed in patients with COVID-19, mainly due to its pro-inflammatory nature and vascular and endothelial involvement. A randomized trial, the HEP-COVID, recruited patients with COVID-19 with DD levels more than 4 times the upper limit of normal and randomized them to receive low-molecular-weight heparin (LMWH) at prophylactic doses vs therapeutic doses. The group of patients with COVID-19 receiving full-dose anticoagulation had a VTE incidence of 10.9% vs 29.0% in the group of patients receiving prophylactic-dose anticoagulation (relative risk [RR] 0.37; 95% CI, 0.21-0.66; p<0.001). Furthermore, patients receiving full-dose anticoagulation had lower mortality rates (19.4%) than those receiving prophylactic-dose anticoagulation (25.0%), (RR 0.68; 95% CI, 0.49-0.96; p=0.03).^25^

Some studies and meta-analyses have shown an association between elevated DD and COVID-19 mortality, whose pathophysiologic mechanism is associated with a secondary state of fibrinolytic hyperactivity or exacerbated fibrin consumption.^26^ A meta-analysis published in 2021 found an association between elevated DD and mortality in hospitalized patients with COVID-19 with lower probability of discharge and greater progression to orotracheal intubation and mechanical ventilation, showing that elevated DD levels on admission would indicate an increased pro-inflammatory state, with maximal activation of the interleukin cascade and a state of hyperfibrinolysis.^27^ Another meta-analysis from 2021 evaluated 10,491 patients with COVID-19 and showed a strong association between elevated DD levels and poor prognosis of the disease (OR 3.39; 95% CI, 2.66-4.33; p<0.00001).^28^ In short, elevated DD levels on the admission of patients with COVID-19 are associated with poor prognosis, increased risk of VTE, progression to mechanical ventilation, and death. There is no reference threshold for DD levels; however, some studies suggest that levels above 2.0 μg/mL on admission could be a predictor of poor prognosis in patients with COVID-19 requiring hospitalization.^29^

In summary, serum DD levels in patients with COVID-19 play a critical role in evaluating coagulation status, inflammation, and risk of complications such as thrombosis and disease severity. However, the results should be interpreted considering the individual clinical setting and other clinical markers, and clinical decision-making should be guided by experienced health care professionals.

#### DD and PTS

PTS is one of the most harmful complications of lower extremity DVT due to its morbidity, with an impact on patients’ quality of life and socioeconomic activities. Despite being a predictor of the inflammatory process and thrombotic activity, DD levels in the follow-up of patients with VTE are not associated with the development of PTS, as documented in several studies in the literature.^30,31^

Residual thrombus detected on CDU, partial or absent venous recanalization, type of anticoagulant administered during DVT treatment, popliteal reflux, and advanced age remain the main determining risk factors for PTS.

In summary, DD plays a key role in the diagnosis, monitoring, and assessment of VTE risk in various clinical settings. DD levels can be influenced by different factors, such as age and underlying medical conditions, and their prognostic value varies depending on the clinical scenario. Therefore, proper interpretation of DD test results is critical to guide the diagnosis and treatment of patients with VTE and other medical conditions.

### Imaging

#### CDU

Venous CDU is the most commonly used complementary method for diagnosing DVT in symptomatic patients. It is less accurate in distal veins, in upper extremity veins, and in asymptomatic patients.^7,9,32,33^ It is currently the imaging modality of choice for diagnosing DVT,^7,9,34^ with sensitivity of 96% and specificity of 98% to 100%,^35^ replacing venography.^7,32,35,36^

Real-time ultrasonography is used to assess the absence or presence of vein compressibility and intraluminal echogenicity. CDU evaluates venous anatomy and venous flow characteristics, combining real-time imaging and spectral analysis.^7,9,37^ Examination of the entire lower-extremity venous system is preferred over segmental examination or point-of-care ultrasound (POCUS), although the issue is still controversial in certain institutions.^38^

In patients with a high probability WS, negative CDU, and positive DD, CDU should be repeated after 3 to 7 days. In cases of recurrent ipsilateral DVT, the criteria used for diagnosis by CDU are as follows: increase in diameter of the same affected segment ≥ 4 mm, increase of 9 cm in thrombus length, or occurrence in a venous segment other than that previously affected.^36^ CDU can distinguish between non-vascular pathologic processes, such as inguinal adenopathy, Baker’s cyst, abscess, and hematoma, among others.

In the follow-up of patients diagnosed with acute DVT, the 2018 Society of Radiologists in Ultrasound consensus recommends using the term “chronic post-thrombotic change” instead of “chronic DVT” to avoid confusion with current DVT. It also recommends caution when using the term “subacute DVT” due to the difficulty in characterizing it.^39,40^

CDU plays a key role in the diagnosis of DVT, providing high sensitivity and specificity. It is also useful in the follow-up of patients with acute DVT, allowing differentiation from other non-vascular conditions. The recommended terminology for describing chronic post-thrombotic changes is intended to avoid confusion in medical communication.

CDU combined with serum DD measurement is commonly used for the diagnosis and confirmation of DVT. The sensitivity and specificity of this combination may vary depending on clinical circumstances, diagnostic criteria, and DD cutoff levels. However, in general:

1. Sensitivity: CDU combined with DD measurement is known to have high sensitivity. This means that most DVT cases are correctly identified by this approach. Sensitivity may vary, but many studies report sensitivity greater than 90% for the diagnosis of DVT;
2. Specificity: The specificity of the CDU and DD combination is generally high but may be lower than the sensitivity. This is because elevated DD levels can occur in several medical conditions other than DVT, such as infections, inflammation, and chronic diseases. Therefore, although specificity may vary, values above 80% are commonly found for this combination.

It is important to highlight that the DD cutoff levels can affect the sensitivity and specificity of the approach. Lower cutoff levels will increase sensitivity but may decrease specificity, whereas higher cutoff levels will increase specificity but may decrease sensitivity.

### Recommendation 3

Moderate or low probability WS associated with normal serum DD levels may preclude the need for CDU to exclude the diagnosis. Class II, level C.^23^

### Recommendation 4

High probability WS associated with elevated serum DD levels suggest the need for CDU to confirm the diagnosis. Class II, level C.^23^

#### Venography/phlebography

Phlebography is considered the gold standard for diagnosing DVT and is currently used only when other tests are unable to define the diagnosis. However, it is not the routine test for suspected DVT due to several limitations, such as high cost, adverse reactions to contrast agents, discomfort for the patient, contraindication for patients with renal failure, and use of radiation. It has limited accuracy in cases of recurrent DVT.^1^ Phlebography may be useful in cases where CDU suggests the presence of proximal obstruction by demonstrating loss of flow phasicity, but the thrombus cannot be visualized in the iliac veins.^41^

#### Computed tomography angiography (CTA)

Because the sensitivity and specificity of CTA are similar to those of CDU, there is insufficient evidence to recommend it as an initial diagnostic modality for DVT.^36^ In a meta-analysis, Thomas et al.^42^ found a sensitivity of 96% (95% CI, 93-98%) and a specificity of 95% (95% CI, 94-97%) for CTA in the diagnosis of proximal DVT in patients with suspected PE. It may be useful for patients with suspected DVT, in whom CDU cannot be used due to technical limitations or suspected venous anomaly.^36^

The use of CTA has the advantage of imaging the pelvic veins and inferior vena cava (IVC), whose imaging is limited in CDU, in addition to detecting other etiologies of lower extremity edema, such as pelvic neoplasms or extrinsic compression by other vascular structures, including the compression of the left common iliac vein (May-Thurner syndrome).^43^ Peterson et al.^44^ demonstrated that, although the sensitivity is high (93%), producing a negative predictive value of 97%, the ability of venous CTA to diagnose DVT has a specificity of 71%, giving a positive predictive value of only 53%; other researchers have shown a 50% false-positive rate for venous CTA in pelvic DVT.

#### Magnetic resonance angiography (MRA)

MRA can be used to diagnose DVT in cases where CDU provides inconclusive results.^36,45^ The accuracy is similar to that of CDU in the diagnosis of DVT of the iliocaval segment.^42,46^ MRA with direct thrombus imaging, based on the paramagnetic properties of methemoglobin, may be the imaging modality of choice for suspected acute recurrent DVT, distinguishing between an old and a new event.^36^

Studies comparing MRA vs phlebography found sensitivity and specificity of up to 100% for MRA in the diagnosis of femoropopliteal DVT, but accuracy was lower for distal DVT. For proximal DVT, MRA has an accuracy similar to that of CDU and phlebography, with the advantage of high detection rates of proximal extension of the thrombosis and isolated pelvic DVT.^47^

Despite excellent accuracy for detecting acute DVT, MRA has several disadvantages. It requires patients to remain still for long imaging times and cannot be used in patients with metal devices or clips. The contrast used (gadolinium) can be toxic in patients with renal dysfunction, and the need for frequent imaging may lead to institutional issues in larger facilities. Finally, MRA quality depends on the institution’s technical experience.^47^

In summary, venography/phlebography is the gold standard but reserved for cases in which other tests do not provide a diagnosis. CTA has sensitivity and specificity similar to those of CDU, while MRA is useful in cases of inconclusive CDU results and can distinguish between an old and a new event in recurrence. The choice of modality depends on patients’ clinical circumstances and technical and safety limitations. CTA or MRA (compared with venography) can be performed when it is not possible to perform CDU.

## TREATMENT

### Anticoagulation in the acute event (conventional patients)

The treatment of DVT poses a challenge and is based on the use of anticoagulants to prevent thrombus progression while the activation of primary fibrinolytic mechanisms promotes its dissolution.^48-51^ The choice of initial anticoagulant includes availability, familiarity of use by the medical team, pharmacokinetic and dynamic characteristics, patient comorbidities, ease of reversing its effect, and even patient preference and cost.^52^ Currently, unfractionated heparin (UFH), LMWH, fondaparinux, vitamin K antagonists (VKAs), and direct oral anticoagulants (DOACs) are available for use in Brazil.

No anticoagulant is completely safe with respect to bleeding. Therefore, in clinical practice, the risk of bleeding should always be assessed when treating VTE. Different anticoagulants may present different risks for bleeding depending on the intensity of treatment, concomitant use of other anticoagulants, thrombolytics, or antiplatelets, and patient characteristics and comorbidities.^53^

### Recommendation 5

In patients with proximal DVT, the preferred approach is generally to use anticoagulant therapy as primary treatment rather than combining it with thrombolytic therapy. Class II, level C.^11,53,54^

#### Duration of anticoagulation

The goal of prolonging treatment duration is to prevent DVT recurrence. The risk is decreased if DVT occurs in the presence of reversible risk factors, such as surgery, and increased if DVT is idiopathic or associated with cancer. Patients with symptomatic PE also have a higher risk of recurrence than those with DVT alone. The risk also increases in the presence of other complications, such as homozygous inherited thrombophilia, APS, or a combination of thrombophilias.^53^

#### Initial anticoagulation of acute lower extremity DVT

For patients with a high clinical suspicion of DVT, it is recommended to start treatment with anticoagulants while awaiting confirmation of the diagnosis, as long as there is no contraindication.^54^ In most patients with proximal DVT, anticoagulant therapy alone is suggested rather than in combination with thrombolytic therapy (conditional recommendation, low-certainty evidence), as shown in Table 1.

#### Notes

Patients with the leg threatened by DVT may require thrombolysis. Using DOACs instead of VKAs is suggested in patients with VTE (conditional recommendation, moderate-certainty evidence) (Table 2).

**Table 2:** DOACs compared with VKAs for VTE.

Outcome	Relative risk (95% CI)	Risk with VKAs	Risk difference with DOACs
Mortality	0.99 (0.85-1.15)	39 per 1000	Null event
Pulmonary embolism	0.97 (0.77-1.23)	20 per 1000	1 fewer per 1000
Deep vein thrombosis	0.80 (0.59-1.09)	26 per 1000	5 fewer per 1000
Major bleeding	0.63 (0.47-0.84)	17 per 1000	6 fewer per 1000

DOAC = direct oral anticoagulant; VKA = vitamin K antagonist; VTE = venous thromboembolism. Adapted from the American Society of Hematology 2020 guidelines.^11^

The recommendation does not apply to all patients, such as to patients with triple-positive APS – when DOACs are contraindicated.

One DOAC is not suggested over another.

We recommend, with a high level of evidence, initial treatment with subcutaneous (SC) LMWH, intravenous (IV) or monitored SC UFH, fixed-dose SC UFH, or SC fondaparinux, followed by long-term oral anticoagulation.

The initial treatment (UFH, LMWH, or fondaparinux) should last for at least 5 days, combined with VKAs from the first treatment day, until the international normalized ratio (INR) is at a therapeutic level (between 2.0 and 3.0) for 2 consecutive days, when parenteral drugs can be safely discontinued. We do not recommend, under any circumstances, initial treatment of DVT with VKAs alone, as they are associated with high rates of symptomatic recurrence and risk of skin necrosis. An alternative to VKAs is the use of DOACs, such as edoxaban and dabigatran, starting on day 5 after heparinization, or apixaban or rivaroxaban without the need for an initial parenteral anticoagulant bridge.

#### LMWH

LMWHs have predictable bioavailability; therefore, routine monitoring with anti-factor Xa level measurements is not recommended.^42,45^ However, blood tests with platelet counts should be performed. They can be administered once or twice daily, in hospital or at home.

#### LMWH vs warfarin

Long-term treatment (more than 35 days) with LMWH is as effective as with warfarin in preventing deaths and VTE recurrence, with a similar risk of bleeding.

#### LMWH vs UFH

In prolonged treatment (3 to 6 months), compared with UFHs, LMWHs are associated with a lower risk of major bleeding and recurrence of proximal DVT, with fewer adverse effects. In patients with acute PE, LMWHs are as effective and safe as UFHs. In patients with acute DVT and severe renal failure, treatment with UFH rather than LMWH is suggested.

#### IV UFH

If IV UFH is the initial treatment of choice, we recommend that, after the IV bolus (80 U/kg or 5000 U), it be administered via continuous infusion (initially at a dose of 18 U/kg/h or 1300 U/h) with dose adjustment to achieve and maintain an activated partial thromboplastin time (aPTT) between 1.5 and 2.5 above baseline.

#### SC UFH

If fixed-dose SC UFH is used, we recommend an initial dose of 333 U/kg followed by 250 U/kg twice daily, with treatment according to the patient’s body weight, with dose adjustment to achieve and maintain an aPTT between 1.5 and 2.5 above baseline.

#### LMWH vs fondaparinux

Fondaparinux is noninferior to LMWH in terms of VTE recurrence, major bleeding, or death.

#### VKAs

VKAs should be initiated on the first treatment day, combined with UFH, LMWH, or fondaparinux, except in patients who have contraindications to their use. Treatment with VKAs requires frequent INR testing and monitoring of food and drug interactions.

#### Warfarin

Achieving a therapeutic INR level (between 2.0 and 3.0) as soon as possible is important, as it reduces the duration of parenteral anticoagulation and costs. Although a dose of 5 mg tends to prevent excessive anticoagulation, an initial dose of 10 mg may more rapidly achieve a therapeutic INR level.

In a systematic review evaluating the effectiveness of warfarin at an initial dose of 10 mg compared with 5 mg in patients with VTE, no differences were observed between the two doses in terms of VTE recurrence, minor or major bleeding, or length of hospital stay. There is no advantage of gradual over abrupt withdrawal of warfarin in terms of preventing VTE recurrence.

#### DOACs

In recent years, 4 DOACs – rivaroxaban, edoxaban, apixaban, and dabigatran – have been made available on the Brazilian market, with some advantages over VKAs, such as fixed dose, fewer drug and food interactions, rapid onset of action, short half-life, and no need for regular monitoring.^55,56^

#### Rivaroxaban

Rivaroxaban is a molecule, with a molecular weight of 436 g/mol.^20^ Its mechanism of action consists of direct inhibition of factor Xa.^57^ The drug is rapidly absorbed orally, reaching maximum plasma concentration 2 to 4 hours following its administration, with a half-life of 6 to 7 hours.^56,57^ Rivaroxaban is metabolized in the liver, and its use should be avoided in individuals with moderate-to-severe liver disease.^56,58^ It is excreted through the kidneys,^10,13^ and its dose should be adjusted in patients with chronic kidney disease (CKD) and creatinine clearance (CrCl) between 15 and 50 mL/min – its use is not recommended with CrCl < 15 mL/min.^13^ Rivaroxaban interacts with P-glycoprotein and CYP3A4 inhibitors.^59,60^

The Einstein DVT study showed that rivaroxaban is as effective as LMWH and warfarin in treating DVT in terms of recurrence rates (2.1% vs 3.0%; p<0.001 for noninferiority), with the same risk of complications. The Einstein EXTENSION and Einstein CHOICE studies also demonstrated the feasibility of its use, with a low risk of major bleeding with prolonged use (up to 12 months). The usual regimen without renal failure is 15 mg orally every 12 hours for the first 21 days, followed by 20 mg orally once daily for the intended treatment duration.^56,61,62^ There is no need for concomitant parenteral drug initially. Its anticoagulant effect can be reversed by andexanet alfa.^57,61,62^

#### Edoxaban

Edoxaban is an oral anticoagulant that directly inhibits factor Xa.^63,64^ It is predominantly absorbed from the duodenum and proximal jejunum,^65-67^ and can be administered with food.^68,69^ It is absorbed within 1 to 2 hours, with a half-life of 5.8 to 10.7 hours.^70-75^ The recommended dose is 60 mg once daily, starting after 5 days of treatment with UFH or LMWH.^76,77^ Its use is contraindicated in patients with CrCl < 15 mL/min.^78^ Edoxaban has potential for use in children (considering preclinical phase I studies).^79,80^

The Hokusai-VTE study showed that, after initial treatment with heparin, edoxaban was noninferior to standard heparin and warfarin therapy.^74,81^ Edoxaban was more effective than warfarin in patients aged ≥ 75 years and in those with multiple comorbidities.^74,75^ Edoxaban has also been shown to be effective and safe in the treatment of cancer-associated VTE.^80,82^ Its dose should be reduced to 30 mg once daily in patients with moderate loss of renal function (CrCl of 15-50 mL/min), weighing less than or equal to 60 kg, or using P-glycoprotein inhibitors.

#### Apixaban

Apixaban is an oral anticoagulant, a direct factor Xa inhibitor. It was approved for medical use in Brazil in July 2011 for the treatment and secondary prevention of VTE.^83-85^ It is predominantly absorbed from the duodenum and proximal jejunum.^86-88^ Maximum plasma concentration is achieved 3 to 4 hours following its administration, with a half-life of 6 hours.^86,87,89-91^ Laboratory monitoring is not required.^92,93^ For treatment in the acute phase of VTE, a dose of 10 mg orally twice daily for 5 days is recommended, followed by 5 mg twice daily.^91,94,95^ There is no need for concomitant use of parenteral anticoagulants. For extended VTE treatment beyond 6 months of initial anticoagulation, a dose of 2.5 mg orally twice daily can be used to reduce recurrence.^87,96-98^ There are no adequate data on its use in pregnant and breastfeeding women.^99^ Safety and efficacy have not yet been established in patients aged < 18 years.^87,98-100^ It is not recommended for use in patients with severe liver failure.^87^ Dose adjustment based solely on renal function is not required in the treatment of VTE, but it is contraindicated in patients with CrCl < 15 mL/min.^87,100^

#### Dabigatran

Dabigatran etexilate is a reversible inhibitor of the thrombin active site. Laboratory monitoring is not required. Its use is not recommended for patients with renal failure with CrCl < 30 mL/min. Like other DOACs, it is contraindicated in patients with advanced liver disease.^101^

The RE-COVER, RE-COVER II, RE-MEDY, and RE-SONATE studies observed noninferiority of dabigatran to standard warfarin therapy in the treatment of PE (fatal or nonfatal), PE, and/or recurrent DVT after an initial course of 5 to 10 days of parenteral anticoagulation. Maximum plasma concentration is achieved 0.5 to 2 hours following its administration, with peak plasma concentration after 6 hours and a half-life of 11 hours. Bleeding is the most important side effect. Its action can be reversed by idarucizumab. Additionally, dabigatran can be cleared by hemodialysis.

Concomitant use with P-glycoprotein inducers should be avoided. It should be initiated after at least 5 days of parenteral anticoagulation at a dose of 150 mg orally every 12 hours throughout the treatment period.^101,102^

### Recommendation 6

The preferential use of DOACs instead of VKAs can be recommended in the initial treatment of VTE. Class II, level B^11,53,54^

#### Uncomplicated, unprovoked VTE in healthy patients

Home anticoagulation, preferably with DOACs, without the need for hospital admission. After the initial treatment of unprovoked DVT or PE, an indefinite period of anticoagulation is suggested (conditional recommendation, moderate-certainty evidence), as shown in Table 3.

**Table 3:** Indefinite anticoagulation compared with discontinuation of anticoagulation in patients with unprovoked VTE after initial treatment.

Outcome	Relative risk (95% CI)	Risk with discontinuation of anticoagulation	Risk difference with indefinite anticoagulation
Mortality	0.75 (0.49-1.13)	18 per 1000	5 fewer per 1000
Pulmonary embolism	0.29 (0.15-0.056)	29 per 1000	21 fewer per 1000
Deep vein thrombosis	0.20 (0.12-0.34)	63 per 1000	50 fewer per 1000
Major bleeding	2.17 (1.40-3.35)	5 per 1000	6 more per 1000

VTE = venous thromboembolism. Adapted from the American Society of Hematology 2020 guidelines.^11^

#### Notes

It does not apply to patients at high risk of bleeding.

For patients with DVT and/or PE who will continue to receive a DOAC for secondary prevention, the use of a standard-dose DOAC or reduced-dose DOAC (rivaroxaban 10 mg/day or apixaban 2.5 mg twice daily) is suggested.

Conditional recommendation, moderate-certainty evidence, Table 4.

**Table 4:** Reduced-dose DOACs compared with standard-dose DOACs in patients who continue anticoagulation indefinitely.

Outcome	Relative risk (95% CI)	Risk with standard dose	Risk difference with reduced dose
Mortality	0.68 (0.10-4.57)	6 per 1000	5 fewer per 1000
Pulmonary embolism	1.25 (0.54-2.91)	5 per 1000	21 fewer per 1000
Deep vein thrombosis	0.75 (0.36-1.53)	9 per 1000	50 fewer per 1000
Major bleeding	0.97 (0.34-2.80)	4 per 1000	6 more per 1000

DOAC = direct oral anticoagulant. Adapted from the American Society of Hematology 2020 guidelines.^11^

For patients with DVT and/or PE with stable cardiovascular disease using acetylsalicylic acid (ASA), discontinuation of ASA is suggested, if possible, during the anticoagulation period.

Conditional recommendation, very low-certainty evidence, Table 5.

**Table 5:** Discontinuation of ASA compared with its continuation (ASA + anticoagulation).

Outcome	Relative risk (95% CI)	Risk with discontinuation of anticoagulation	Risk difference with continuation of ASA combined with anticoagulation
Major bleeding	1.26 (0.34-2.80)	29 per 1000	6 more per 1000 (2 fewer per 21 more)

ASA = acetylsalicylic acid. Adapted from the American Society of Hematology 2020 guidelines.^11^

Note: it does not apply to patients with a recent coronary event or coronary intervention.

#### Treatment duration

For initial treatment of VTE, anticoagulation for 3 to 6 months rather than long-term anticoagulation (6 to 12 months) is suggested.

Conditional recommendation, moderate-certainty evidence, Table 6.

**Table 6:** Long-term compared with short-term anticoagulation for patients with VTE provoked by a transient risk factor.

Outcome	Relative risk (95% CI)	Risk with short-term anticoagulation	Risk difference with long-term anticoagulation
Mortality	1.38 (0.85-2.23)	18 per 1000	7 fewer per 1000
Pulmonary embolism	0.66 (0.29-1.151)	50 per 1000	17 fewer per 1000
Deep vein thrombosis	0.50 (0.29-0.95)	117 per 1000	50 fewer per 1000
Major bleeding	1.46 (0.78-2.73)	13 per 1000	6 more per 1000

VTE = venous thromboembolism. Adapted from the American Society of Hematology 2020 guidelines.^11^

Note: for VTE provoked by a transient risk factor, secondary prevention does not need to be considered.

#### Provoked VTE

For patients who develop VTE provoked by a transient risk factor without a history of provoked VTE, discontinuation of anticoagulation is suggested after completion of the initial treatment.

Conditional recommendation, moderate-certainty evidence, Table 7.

**Table 7:** Long-term compared with short-term anticoagulation for patients with recurrent provoked VTE.

Outcome	Relative risk (95% CI)	Risk with short-term anticoagulation	Risk difference with long-term anticoagulation
Mortality	0.75 (0.49-1.13)	18 per 1000	7 fewer per 1000
Pulmonary embolism	0.29 (0.15-0.056)	29 per 1000	17 fewer per 1000
Deep vein thrombosis	0.20 (0.12-0.34)	117 per 1000	50 fewer per 1000
Major bleeding	2.17 (1.40-3.35)	5 per 1000	6 more per 1000 (3 fewer per 12 more)

VTE = venous thromboembolism. Adapted from the American Society of Hematology 2020 guidelines.^11^

For patients with PE with echocardiography and/or biomarkers compatible with right ventricular dysfunction but without hemodynamic compromise (submassive PE), anticoagulation alone is suggested rather than the routine use of thrombolysis plus anticoagulation.

Conditional recommendation, low-certainty evidence.

Note: thrombolysis is reasonably considered for younger people with submassive PE with low bleeding risk.

For patients with VTE who will continue to receive secondary prophylaxis, the use of anticoagulation over ASA is suggested.

Conditional recommendation, moderate-certainty evidence, Table 8.

**Table 8:** ASA compared with anticoagulation for patients receiving secondary prophylaxis for prior VTE.

Outcome	Relative risk (95% CI)	Risk with anticoagulation	Risk difference with ASA
Mortality	0.86 (0.31-2.35)	7 per 1000	1 fewer per 1000 (5 fewer per 10 more)
Pulmonary embolism	3.10 (1.24-7.73)	5 per 1000	11 more per 1000
Deep vein thrombosis	3.15 (1.50-6.63)	8 per 1000	17 more per 1000
Major bleeding	0.49 (0.12-1.95)	5 per 1000	3 fewer per 1000

ASA = acetylsalicylic acid; VTE = venous thromboembolism. Adapted from the American Society of Hematology 2020 guidelines.^11^

### Recommendation 7

For the initial treatment of VTE associated with a major transient risk factor and a history of recurrent VTE, anticoagulation for 3 to 6 months is recommended, rather than long-term treatment, which extends from 6 to 12 months. Class II, level B.^11,53,54^

### Recommendation 8

In patients with recurrent VTE associated with a persistent risk factor, indefinite oral anticoagulation is recommended. Class I, level B.^103^

### Recommendation 9

In patients experiencing their first episode of VTE, with an unidentifiable or persistent risk factor and a low risk of bleeding, secondary prophylaxis with anticoagulants should be considered. Class IIa, level A.^103-111^

### Recommendation 10

After initial treatment of unprovoked VTE, an indefinite anticoagulation regimen is recommended. For patients who will continue to receive a DOAC for secondary prevention, the use of a standard-dose DOAC or reduced-dose DOAC (eg, rivaroxaban 10 mg/day or apixaban 2.5 mg twice daily) is suggested. Class II, level B.^11,53,54^

### Recommendation 11

In patients with an indication for secondary prophylaxis for non-cancer-associated VTE, the use of low-dose DOACs (rivaroxaban 10 mg/day or apixaban 2.5 mg every 12 hours) is recommended over higher-dose DOACs, dicoumarins, or ASA. Class IIa, level A.^110,111^

### Recommendation 12

In patients receiving secondary prophylaxis for VTE, the use of anticoagulation is recommended over the use of ASA. Class II, level B.^11,53,54^

### Recommendation 13

In patients with an indication for secondary prophylaxis for VTE and APS, secondary prophylaxis with the use of dicoumarins is recommended. Class I, level A.^112,113^

### Anticoagulation in acute VTE in CKD

CKD is a paradoxical disease in the treatment of VTE because, while it increases the risk of recurrence due to endothelial dysfunction, initial platelet hyperreactivity, increased fibrin formation, and decreased fibrinolytic activity, it also increases the risk of major bleeding, with progressive worsening of the disease due to decreased platelet aggregation.^114^

This delicate balance between the risk of recurrence and bleeding makes VTE treatment a challenge especially in those with moderate-to-severe CKD (CrCl between 15 and 59 mL/min) or end-stage CKD (CrCl < 15 mL/min).^114^

Patients with CKD and VTE are traditionally treated with IV UFH followed by warfarin, with a target INR between 2.0 and 3.0.^115^ For patients with VTE and end-stage CKD (CrCl < 15 mL/min), LMWHs, fondaparinux, and DOACs are not recommended.^115,116^ In moderate-to-severe CKD (CrCl between 15 and 59 mL/min), LMWHs or fondaparinux may eventually be prescribed with caution, in an adjusted dose, with monitoring of anti-factor Xa levels, or not recommended to avoid bioaccumulation and bleeding, particularly in advanced CKD (CrCl < 30 mL/min).^115,117^

The efficacy and safety of DOACs, with possible dose adjustment, were confirmed in the initial and extended treatment of VTE in patients with VTE and mild-to-moderate CKD (CrCl between 30 and 80 mL/min).^115^ Edoxaban is the only DOAC with a recommendation for dose reduction in the acute treatment of VTE for patients with CKD (CrCl between 30 and 50 mL/min), from 60 to 30 mg orally once daily, while maintaining the same efficacy and safety.^118^

Regular reassessment of renal function is essential in these patients.

### Anticoagulation in acute VTE in women

In high-income countries, where the classic causes of maternal death during pregnancy have been controlled, such as eclampsia and bleeding, VTE has become the main concern, and its prevention, supported by evidence-based guidelines with the use of pharmacological prophylaxis in selected patients, is still the best strategy to reduce this potentially fatal complication.

The choice of contraceptive methods and hormone replacement therapy (HRT) should be made through careful selection by evaluating eligibility criteria, contraindications, and patient autonomy. Both indiscriminate use and unreasonable prohibition are inappropriate approaches. Hormonal contraceptives and HRT increase the risk of VTE; however, women should not be deprived of its benefits, in addition to contraception, which give women more freedom during their childbearing years and make them less symptomatic during perimenopause.

Assisted reproduction and hormone therapy (HT) in transgender women are also associated with the development of VTE, and its prevention will depend on correct indication and adequate prophylaxis in those patients considered at high risk for developing this disease.

#### VTE in the pregnancy-puerperal cycle

Pregnant women have all 3 etiopathogenic components of Virchow’s triad: a) venous stasis, caused by compression of the IVC and left common iliac vein by the pregnant uterus and by reduced venous tone resulting from the myorelaxant action of progesterone; b) hypercoagulability, secondary to the induction of hepatic synthesis of coagulation factors VII, VIII, and X by placental estriol, increased levels of fibrinogen and plasminogen activator inhibitors I and II, and reduced protein S synthesis; and c) endothelial injury, which occurs during nidation, endovascular remodeling of the uterine spiral arteries, and expulsion of the placenta.^119,120^

During pregnancy, there is a 5- to 10-fold increase in the risk of VTE, and it can be 35 times higher in the puerperium compared with the rate among non-pregnant women of the same age. After childbirth, its prevalence decreases rapidly; but there is a residual risk for up to about 12 weeks. Approximately two-thirds of DVTs occur during pregnancy, distributed equally across the 3 trimesters. However, 43% to 60% of PE episodes occur in the first 6 weeks postpartum.^119,120^

Among pregnant women, compared with non-pregnant women, DVTs are even more prevalent in the left lower extremity (90% vs 55%) and in the iliofemoral segment (72% vs 9%). This is due to the marked compression of the vena cava and left common iliac vein against the fifth lumbar vertebra, caused by the growing pregnant uterus.^119,120^

The main risk factors for VTE during pregnancy and puerperium are as follows: overweight; obesity; age 35 years or over; inherited or acquired thrombophilia; long-distance travel; immobility; hospital admission during pregnancy; multiparity; smoking; comorbidities, such as inflammatory bowel disease; urinary tract infection; systemic lupus erythematosus; pregnancy-induced hypertension or preeclampsia; non-obstetric antepartum surgery; bleeding; blood transfusion; and hyperemesis gravidarum; among others. Prevention of VTE in pregnancy, through the application of evidence-based guidelines or risk assessment models and the implementation of mechanical and/or pharmacological prophylaxis, is still the best strategy to reduce the rate of these events.^119,120^

Peculiarities of anticoagulant therapy during pregnancy and puerperium. Administration of warfarin at 6 to 12 weeks’ gestation can induce fetal embryopathy (nasal hypoplasia and/or stippled epiphyses), central nervous system abnormalities (dorsal midline dysplasia with agenesis of the corpus callosum, midline cerebellar atrophy, ventral midline dysplasia with optic atrophy and amaurosis, and bleeding), and fetal bleeding.^121^ However, warfarin is safe and can be prescribed during breastfeeding.^121^ DOACs are contraindicated in pregnancy, because they cross the placental barrier, and in breastfeeding, because they pass into breast milk.^121^ Therefore, VTE should be treated during these periods preferably with UFH, or with LMWH if there are restrictions on the use of UFH.^122^

The use of fondaparinux during pregnancy has a restricted indication and low-quality evidence – the American College of Chest Physicians (ACCP) recommendation was generally grade 2C, indicating a moderate grade of recommendation based on low-quality evidence or consensus opinion of experts.^54,84^

### Recommendation 14

During pregnancy and breastfeeding, we recommend against the use of DOACs. For the treatment of VTE during pregnancy, UFH or LMWH are recommended. Class I, level B (UFH x LMWH) and Class I, level A (LMWH x VKA)^54,84,121,122^

#### Choice of mode of delivery in women on anticoagulation therapy

The choice of the mode of delivery is obstetric. There is no contraindication to artificial cervical ripening or labor induction. The delivery of a pregnant woman receiving anticoagulation should be scheduled at 37 to 40 weeks’ gestation.^123^ LMWHs should be discontinued 12 hours before delivery if administered at prophylactic doses, or 24 hours before delivery if administered at intermediate or therapeutic doses, thus allowing the safe administration of spinal or epidural anesthesia.^123,124^

Regardless of the pharmacological prophylaxis and the chosen mode of delivery, pregnant women should wear elastic compression stockings throughout the procedure.^4^ Although the risk associated with cesarean delivery alone is low, the incidence of VTE increases in the presence of other associated risk factors. Therefore, thromboprophylaxis should be prescribed based on the risk stratification of each pregnant woman, with a validated risk assessment model.^123,124^

#### VTE and contraception

In Brazil, 1 in every 5 women uses oral contraceptives (OCs), which offer benefits beyond contraception, such as reduced menstrual bleeding, dysmenorrhea, premenstrual syndrome, migraines, acne, and hirsutism. OCs also have long-term benefits, such as reduced incidence of endometrial, ovarian, and colorectal cancer.^122^

OCs increase the risk of VTE from an initial rate of 5 per 10,000 woman-years among nonusers to 9-10 per 10,000 woman-years among users. To keep this risk in perspective, it is important to note that the risk of VTE is 29 per 10,000 women during pregnancy and 300-400 per 10,000 women during puerperium.^122^

The thromboembolic risk of OCs is related to the dose of estrogen and the type of progestin combined with it. Older OCs with high estrogen levels (> 50 μg ethinyl estradiol) are associated with a higher risk of VTE than newer OCs (< 50 μg ethinyl estradiol). However, no risk reduction was confirmed with OCs containing 20 μg of ethinyl estradiol compared with those containing 30 μg of ethinyl estradiol.^122^ The type of progestin also influences the risk of VTE, where second-generation progestins (levonorgestrel [LNG] and norethisterone) are safer than third- and fourth-generation progestins.^122^

The OC-related risk of VTE is associated with increasing body weight and age and with the reintroduction or change of OC after discontinuation exceeding 4 weeks.

Among OC users, those with inherited thrombophilia are at increased risk of developing VTE. However, given the low prevalence of inherited thrombophilia in the general population and the high cost of diagnostic laboratory screening, routine screening is not recommended.^122^ Personal or family history of VTE is the most important and most common risk factor for OC-associated VTE.^122^ Non-oral contraceptives, including transdermal patches and vaginal rings, are also associated with an increased risk of VTE, increasing the risk by 7.9 and 6.5 times, respectively.^122^ Coagulation does not show significant changes with the use of progesterone-only OCs, LNG-containing implants, LNG-releasing intrauterine system, or depot medroxyprogesterone acetate injection. Therefore, it can be safely used in this scenario.^122^

#### TEV and assisted reproduction

In vitro fertilization (IVF) is the most widely used technique for human reproduction in infertile couples, and VTE is a rare complication of this technique, occurring in only 0.1% to 2.4% of fertilization cycles.^122^ The risk of VTE doubles in pregnancies after IVF compared with the baseline risk of pregnant women not undergoing IVF. This is particularly due to the 5- to 10-fold increase in risk during the first trimester of pregnancies after IVF, secondary to ovarian hyperstimulation syndrome (OHSS), an iatrogenic and potentially fatal complication that occurs in 33% of all IVF cycles. Women who develop OHSS have a 100 times higher risk of VTE and, in severe OHSS, thromboprophylaxis with LMWH reduces the incidence of VTE without significantly increasing bleeding.^122^

IVF-related VTE occurs most frequently between days 40 and 42 after embryo transfer and tends to be located in the upper extremities and cervical region rather than in the left lower extremity, the most common site in spontaneous pregnancies.^122^ IVF also increases the risk of arterial thrombosis, which occurs on average on day 10 after embryo transfer.^122^

#### VTE and HRT

Although recent data show that the risks of HRT may outweigh its benefits, many women still receive HRT with estrogens, indiscriminately, to minimize menopausal symptoms. This approach is associated with an increased incidence of VTE, especially in the first year of treatment. Additionally, women with a uterus also receive progestins to counteract the risk of endometrial cancer on HRT, which may be an additional risk factor for developing VTE.^122^

Observational studies, systematic reviews, and meta-analyses consistently report a 2- to 3-fold higher risk of VTE in postmenopausal women on HRT than in postmenopausal women not on HRT.^122^

The risk of VTE in women receiving HRT depends on the route of administration.

Oral estrogens cause procoagulant changes, such as increased resistance to activated protein C, by reducing serum protein S levels and fibrinolytic activity, probably because they pass through and are metabolized in the liver; however, these changes are not observed in HRT via the transdermal route. There are no adequate data on the use of transdermal estrogen in pregnant and breastfeeding women.^122^

To prevent VTE in women who will receive HRT, it is important to identify those most susceptible. Those with a personal or family history of VTE are considered to be at high risk and, therefore, are not candidates for HRT with oral estrogens.^122^

HRT is, in fact, the most effective treatment to minimize menopausal symptoms associated with a drop in estrogen levels during menopause. However, it should not be prescribed indiscriminately. After evaluation of the risks and benefits, the best strategy to protect against the risk of VTE is to prescribe HRT with the lowest transdermal estrogen dose possible, alone or combined with micronized progestins, for the shortest duration possible.^123^

#### VTE in transgender women

The terms transgender and gender nonconformity describe a situation in which a person’s gender identity differs from the external sexual anatomy they were born with. The goals of gender affirmation in transgender women are to suppress masculine characteristics and induce feminine characteristics to the extent possible.

The provision of physician-guided gender-affirming HT and surgery supported by the health system has improved quality of life and reduced disorders observed in this population, including VTE.^124^

Several studies have demonstrated an increased risk of VTE in transgender women receiving HT, and this risk is related to the type and dose of the hormones used and, mainly, to their route of administration. Transdermal is the preferred route of administration in transgender women with a personal or family history of VTE and in those with inherited thrombophilia.^124^ It is worth noting that, for this population, HT is not an elective therapy, but rather an absolute necessity to achieve the desired phenotype. In many places, transgender women live on the fringes of society, without access to health professionals or services qualified to prescribe HT. As a consequence, HT is often obtained illegally and taken on one’s own, without professional guidance on safe composition, doses, and routes. Another point to ponder is that non-oral routes of HT administration are usually more expensive than the oral route, thus becoming inaccessible for most patients.^124^

A plausible strategy to mitigate the risk of VTE in at-risk groups is the concomitant initiation of prophylactic anticoagulation with HT, especially for the first 6 to 12 months of treatment.^124^ Adequate prescriptions for contraception and HRT also require maturity and medical knowledge. Simply prohibiting the use of OCs and HRT without carefully assessing each patient’s risk factors and personal or family history does not have a decisive impact on the occurrence of VTE and unnecessarily exposes women to the risk of an unwanted pregnancy and/or a decline in quality of life in any age group.

### Extended anticoagulation – secondary prophylaxis

Secondary VTE prophylaxis, also called “extended therapy,” consists of maintaining antithrombotic therapy (anticoagulants or, less frequently, antiplatelet agents) after the initial period of acute VTE treatment (3 to 6 months), with the purpose of preventing recurrence.

The incidence of a new VTE event after initial treatment can exceed 40% within 10 years.^125^ However, it is possible to identify individuals at higher risk of recurrence and then target extended anticoagulation to patients who will benefit from such a strategy. Two elementary factors should be considered in the decision-making process to indicate secondary VTE prophylaxis: the risk of VTE recurrence and the risk of bleeding in patients receiving anticoagulation. Therefore, the risk of VTE recurrence should be estimated considering the factors that characterize low, intermediate, and high risk, as detailed in Table 1. Patients at low risk of recurrence have no indication for extended secondary prophylaxis.^104,126^ Conversely, patients with recurrent VTE without identifiable risk factors or with persistent risk factors usually benefit from extended therapy.^103^ Finally, patients at intermediate risk of VTE often benefit from secondary prophylaxis as long as they have a low risk of bleeding.^105-107^

Major transient risk factors reduce the likelihood of recurrence, as VTE only occurs in the presence of a strong thrombotic stimulus. The occurrence of VTE in the presence of minor risk factors is associated with an increased risk of recurrence. The rationale behind this evidence is the fact that patients who developed VTE even in the presence of weak stimuli are more prone to recurrence,^105^ as shown in Table 9.

**Table 9:** Risk of recurrent VTE and risk of bleeding associated with the use of anticoagulants.

Risk of recurrent VTE		Risk of bleeding associated with the use of anticoagulants
Risk	Examples	Age > 75 years, previous bleeding without a treatable or reversible cause, active cancer, previous stroke, concomitant use of antiplatelet agents or NSAIDs, chronic kidney disease, chronic liver disease, frail patients, poor anticoagulation control (for the use of dicoumarins).
Low (< 3% per year)	Major transient or reversible risk factors: major surgery, multiple trauma, medical hospitalization.	
Intermediate (3-8% per year)	Minor surgery, inflammatory bowel disease, autoimmune disease, estrogen use, pregnancy/puerperium, thrombosis associated with long-distance travel, thrombophilia (except APS), VTE with no identifiable risk factor.	
High (> 8% per year)	Active cancer, APS, recurrent VTE with no identifiable risk factor.	

VTE = venous thromboembolism; NSAID = nonsteroidal anti-inflammatory drug; APS = antiphospholipid syndrome. Adapted from Konstantinides et al.^127^

Secondary prophylaxis with ASA or low-dose warfarin (target INR between 1.5 and 2.0) did not demonstrate a good efficacy and safety relationship in preventing VTE recurrence.^128-130^

More recently, with the advent of DOACs and their superiority in safety outcomes,^108-111^ new investigations were conducted, validating their use in the initial and extended treatment of VTE. These studies are shown in Table 10. In phase IV studies, DOACs have demonstrated efficacy and safety results comparable to those obtained in phase III randomized clinical trials.^131-141^

**Table 10:** Comparison of safety outcomes between DOACs.

**Study**	**Groups, N**	**Reduced recurrent VTE/death with DOACs**	**Major bleeding (%)**	**Major bleeding + Clin, Relevant %**
RE-SONATE	Dabigatran, 681	-92% (0.08; 0.02-0.25)*	Dabigatran: 0.3	Dabigatran: 5.3
	Placebo, 662		Placebo: 0.0	Placebo: 1.8*
EINSTEIN-EXT	Rivaroxaban, 602	-82% (0.18; 0.09-0.39)*	Rivaroxaban: 0.7	Rivaroxaban: 6.0
	Placebo, 595		Placebo: 0.0	Placebo: 1.2*
AMPLIFY-EXT	Apixaban 2.5, 840	-81% (0.33; 0.22-0.48; A 2.5 mg vs placebo)	Apixaban 2.5: 0.2	Apixaban 2.5: 3.2
	Apixaban 5.0, 813	-80% (0.36; 0.25-0.53; A 5 mg vs placebo)*	Apixaban 5.0: 0.1	Apixaban 5.0: 4.3
	Placebo, 829		Placebo: 0.5	Placebo: 2.7
RE-MEDY	Dabigatran, 1430	1.3% warfarin	Dabigatran: 0.9	Dabigatran: 5.6*
	Warfarin, 1426	1.8% dabigatran	Warfarin: 1.8	Warfarin: 10.2
		(1.44; 0.78-2.64)		
EINSTEIN CHOICE	Rivaroxaban 20 mg, 1121	-66% (0.34; 0.20-0.59; Riva 20 mg vs ASA)*	Riva 20: 0.5	Riva 20: 3.3
	Rivaroxaban 10 mg, 1136	-74% (0.26; 0.14-0.47; Riva 10 mg vs ASA)*	Riva 10: 0.4	Riva 10: 2.4
	ASA 100 mg, 1139		ASA 100: 0.3	ASA 100: 2.0

DOAC = direct oral anticoagulant; VTE = venous thromboembolism; ASA = acetylsalicylic acid.

Dabigatran was superior to placebo and noninferior to warfarin in reducing recurrent VTE. Compared with placebo, dabigatran had more bleeding events, although there was no statistical difference in major bleeding events. Compared with warfarin, dabigatran was superior in reducing clinically relevant bleeding events and noninferior in the incidence of major bleeding.^108^

The efficacy and safety of rivaroxaban were tested in two separate investigations.^1,28,110^ In the first, rivaroxaban at a therapeutic dose (20 mg/day) demonstrated superior efficacy to placebo, but with more bleeding events (no difference in major bleeding). A second three-group study compared two different doses of rivaroxaban (10 and 20 mg/day) vs ASA 100 mg/day. Both doses of rivaroxaban showed similar efficacy in preventing VTE recurrence, and both were superior to ASA. There was no difference in bleeding between the groups.

A study was conducted to determine the efficacy and safety of apixaban in reducing recurrent VTE. Two doses of apixaban (2.5 mg and 5.0 mg every 12 hours) were compared with placebo, both showing superiority in efficacy and noninferiority in safety.^111^ It is important to note that patients at high risk of bleeding were systematically excluded from the aforementioned studies.

#### Dose and choice of antithrombotic agent

Although warfarin and ASA have been superior to placebo in preventing recurrent VTE, subsequent studies have demonstrated the superiority of DOACs for this purpose. Therefore, VKAs and ASA should be used for this purpose only in situations where DOACs are contraindicated, such as in APS.^112^ In the only comparative study between warfarin and rivaroxaban in patients with triple-positive APS, warfarin demonstrated superiority in the composite outcome of efficacy and safety, being the drug of choice for this group of patients.^113^ However, it remains unclear whether all patients should receive reduced doses. In patients at high risk of recurrence and low risk of bleeding, the use of full-dose DOACs appears to be the appropriate choice.

#### Duration of extended VTE treatment

There is no rigid definition of the optimal duration of extended VTE treatment. Most clinical trials investigating this strategy reported a duration of 1 to 2 years, with few exceeding 36 months. However, for patients with recurrent VTE with no identifiable or persistent risk factors, indefinite anticoagulation is recommended.^103^ The term “life-long anticoagulation” should be avoided, since the maintenance of anticoagulant therapy should be regularly reassessed, considering the balance between the risk of VTE recurrence and the risk of bleeding.

### Interventional treatment of acute VTE

#### IVC filter

The use of IVC filters has been associated with numerous controversies in recent years. There are reports of excessive use for questionable indications and failure to retrieve the devices, which were designed to be temporary, with subsequent thrombotic complications. It should be noted that the sole purpose of IVC filters is to prevent PE and, therefore, reduce its morbidity and mortality. However, IVC filters are the only viable treatment option for patients with DVT with contraindication to anticoagulation, although randomized trials are needed.

In the Prévention du Risque d’Embolie Pulmonaire par Interruption Cave (PREPIC) study,^142^ 400 patients with proximal DVT, with or without concomitant symptomatic PE, were randomized to receive a permanent IVC filter or no filter. The study used a two-by-two factorial design, and patients were also randomized to SC LMWH (enoxaparin) or IV UFH (aiming at an aPTT ratio of 1.5 and 2.5). All patients underwent baseline and follow-up perfusion/ventilation lung scanning when symptoms of potential PE occurred, or between 8 and 12 days to assess for asymptomatic PE. The primary outcome was the occurrence of PE (symptomatic or asymptomatic) within 12 days of randomization. A number of symptom-based secondary outcomes were also assessed. On day 12, symptomatic or asymptomatic PE occurred in only 2 patients (1.1%) in the filter group vs 9 patients (4.8%) in the no-filter group. At 2 years, 6 PE events occurred in the filter group (1 death) and 12 in the no-filter group (5 deaths; OR 0.5; 95% CI, 0.19-1.33; p=0.16), but recurrent DVT occurred in 37 patients (20.8%) in the filter group vs 21 patients (11.6%) in the no-filter group (OR 1.87; 95% CI, 1.1-3.2; p=0.02). Mortality rates were similar in both groups at 2 years (43 patients in the filter group vs 40 patients in the no-filter group). The 8-year results were published in 2005 and showed that symptomatic PE occurred in 9 patients (6%) in the filter group vs 24 (15%) in the no-filter group (p=0.008). However, recurrent DVT was more common in the filter group (57 patients vs 41 patients; p=0.042). Despite the reduced risk of PE, the authors concluded, based on the increased risk of recurrent DVT and lack of survival benefit, that the systematic use of IVC filters cannot be recommended for this population.

In the prospective, randomized PREPIC 2 study,^143^ published in 2015, 399 patients with symptomatic PE and DVT were analyzed. Patients with at least one criterion for severity, ie, age > 75 years, active cancer, chronic cardiac or respiratory failure, ischemic stroke with leg paralysis within the last 6 months (but more than 3 days before randomization), DVT involving the iliocaval segment or bilateral DVT, or at least one sign of right ventricular dysfunction or myocardial injury, were assigned to the retrievable IVC filter implantation plus anticoagulation group (n = 200) or the anticoagulation alone group (n = 199). Patients in both groups, with and without IVC filter, received anticoagulation for 6 months, and filter retrieval was planned at 3 months from placement. The filter was successfully inserted in 193 patients and was retrieved as planned in 153 of the 164 patients in whom retrieval was attempted. At 3 months, recurrent PE occurred in 6 patients (3.0%; all fatal) in the filter group and in 3 patients (1.5%; 2 fatal) in the control group (RR with filter, 2.00; 95% CI, 0.51-7.89; p=0.50). Results were similar at 6 months. No difference was observed between the 2 groups regarding the other outcomes. Filter thrombosis occurred in 3 patients. The authors concluded that, among hospitalized patients with severe acute PE, the use of a retrievable IVC filter plus anticoagulation, compared with anticoagulation alone, did not reduce the risk of symptomatic recurrent PE at 3 months. These findings do not support the use of this type of filter in patients who can be treated with anticoagulation.

In the Filter Implantation to Lower Thromboembolic Risk in Percutaneous Endovenous Intervention (FILTER-PEVI) study,^144^ 141 patients undergoing early thrombus removal were randomized to receive an IVC filter or no filter. Patients with and without PE at presentation were included. Only symptomatic patients were investigated, and PE was identified in 1 patient in the filter group and in 8 in the no-filter group. However, there was no difference in mortality between the groups, and the study was weakened by the lack of preoperative imaging of the pulmonary arteries.

Absolute contraindication to anticoagulation includes uncontrollable active bleeding, high risk of major bleeding (eg, coagulation defect, severe thrombocytopenia, recent intracerebral hemorrhage, or brain injury at high risk of bleeding), or urgent surgery requiring discontinuation of anticoagulation.^145^

There appears to be a consensus with good quality evidence that anticoagulation should not be used concomitantly with IVC filter implantation, nor in cases of temporary contraindication to anticoagulation; in patients with an already implanted filter, anticoagulation should be resumed as soon as the risk of bleeding is resolved.^11,54,84,146^

### Recommendation 15

In patients with acute PE, receiving anticoagulation, we recommend against IVC filter insertion. Class II, level B^143-145^

### Recommendation 16

In patients with proximal DVT and a formal contraindication to starting anticoagulation, or up to the first 3 months of treatment, we recommend the insertion of a temporary IVC filter. Class II, level B.^143-145^

### Recommendation 17

In patients with acute PE and an IVC filter inserted as an alternative to anticoagulation, we recommend conventional anticoagulant therapy if the risk of bleeding has been resolved. Class II, level B.^143-145^

#### Interventional treatment of acute VTE – thrombolysis, pharmacomechanical thrombectomy (PMT)

There are many published studies on the interventional treatment of acute VTE, but only 4 prospective randomized studies have sufficient power to analyze the results: CaVenT, ATTRACT, ATTRACT (phase III), and CAVA.^147-153^

The CaVenT study compared adjunctive catheter-directed thrombolysis (CDT) with recombinant tissue plasminogen activator (rt-PA) in addition to anticoagulation alone followed by continued oral anticoagulants in 2 groups for at least 6 months. The primary efficacy outcome was the presence of PTS, defined as a score ≥ 5 on the Villalta scale in the affected extremity or presence of an ulcer in the leg at the 24-month visit. The study showed a significant reduction in PTS in the intervention group, with an absolute risk reduction of 14.4% at 24 months.^147-149^ Long-term follow-up results from the CaVenT study showed that the absolute risk reduction increased to 28% after 5 years. The number needed to treat decreased from 7 to 4,^147^ and no difference was detected in quality of life, but the study did not consider this outcome. Quality of life worsened significantly in patients who developed PTS across the entire study population.^148^ Furthermore, a cost-effectiveness analysis demonstrated a net benefit of treatment, with an increase in the cost-effectiveness ratio of US$20,000 per quality-adjusted life years.^150^

The ATTRACT trial was a prospective, multicenter, randomized trial that evaluated PMT and CDT for the prevention of PTS in patients with femoral or more proximal DVT compared with standard therapy with oral anticoagulants alone.^151^ The protocol consisted of 3 different modalities (CDT alone or combined with PMT using Angiojet®/Trellis-8®) in patients randomized to the treatment group, at the discretion of the treating physician. The primary efficacy outcome was similar to that of the CaVenT study, that is, the presence of PTS, defined as a Villalta score ≥ 5 in the affected extremity or an ulcer, occurring at any time between the post-randomization 6-month follow-up visit and the 24-month follow-up visit (included). Over 24 months, there was no significant between-group difference in the percentage of patients who developed PTS (47% with CDT/PMT vs 48% with standard therapy; RR 0.96; 95% CI, 0.82-1.11; p=0.56). PMT led to more major bleeding events at 10 days (1.7% PMT vs 0.3% standard therapy; p=0.049), but no significant difference in recurrent VTE was observed at 24 months (12.5% PMT vs 8.5% standard therapy; p=0.087). The intervention reduced leg pain and edema at 30 days but did not significantly improve quality of life from baseline to 24 months of treatment. The intervention significantly reduced both PTS severity scores and the development of moderate-to-severe PTS (18% with PMT vs 24% with standard therapy; RR 0.73; 95% CI, 0.54-0.98; 0.04) over the 24-months period.

The ATTRACT (Phase III) trial^152^ was a phase III, multicenter, open-label, assessor-blind, randomized clinical trial. Angiojet® was used for PMT and, in cases where the thrombus extended more distal to the popliteal vein, CDT could be performed prior to PMT. In femoropopliteal DVT, except for reduced PTS at 6 months of follow-up (21.7% with PMT vs 40.8% with control; p=0.01), no significant differences were identified in early or late study outcomes between the PMT and control-arm patients. In iliofemoral DVT, PTS developed in 41 (43.2%) of 95 patients treated with PMT and 40 (40.0%) of 100 control-arm patients (p=0.65) at 24 months.

The point estimates of the Villalta scale and venous clinical severity scale scores from 6 to 24 months and of the proportions of patients with PTS at 6 months (26.2% vs 39.2%; p=0.08) were nominally lower for PMT than for control, but the differences were not statistically significant, nor were the differences observed in the 2-year occurrences of moderate-to-severe PTS, severe PTS, venous ulceration, or quality of life from 6 to 24 months.

Improvement in early disease severity was greater for PMT than for control, reaching statistical significance at 30 days (mean difference of 0.84 Likert scale points; p=0.0061). From baseline to 30 days, patients treated with PMT had greater improvement in quality of life (difference of 12.6 VEINES-QOL scale points; p=0.0001) and symptoms (difference of 11.4 VEINES-Sym scale points; p=0.0003) than the control-arm patients. At 30 days, those treated with PMT also had less residual thrombus. Regarding complications, major bleeding, PE, renal failure, and bradycardia were infrequent with PMT (< 2% each), but 24-month VTE recurrence was more frequent (13.9% with PMT vs 6.8% with control; p=0.03).

The CAVA trial was a prospective, multicenter, randomized trial that compared the use of ultrasound-accelerated thrombolysis with standard anticoagulation alone.^153^ In contrast to the ATTRACT trial, the CAVA trial involved only iliofemoral DVT, thus addressing one of the criticisms raised about the inclusion criteria of the ATTRACT trial. Overall, the CAVA trial randomized 184 patients: 91 to intervention and 93 to standard therapy, with 77 receiving the intervention and 75 remaining in the standard therapy group after screening failure/withdrawal of consent. After 12 months of follow-up, the trial showed no between-group differences, with 22 (29%) of 77 patients who received the intervention vs 26 (35%) of 75 patients who received standard therapy developing PTS (p=0.42). These data led to the conclusion that, for patients with acute proximal DVT, PMT did not prevent PTS but increased major bleeding. The results suggested that there may be a benefit in reducing the risk of moderate-to-severe PTS, despite the higher-than-expected rate of PTS in patients treated with early thrombus removal.^153^ However, no benefit was observed in patients with femoropopliteal DVT.^154^

In a Cochrane systematic review,^155^ comparing thrombolysis strategy with anticoagulation for lower extremity DVT, patients who received thrombolysis had more bleeding episodes (6.7% vs 2.2%) (RR 2.45; 95% CI, 1.58-3.78; 1943 participants, 19 studies; moderate-certainty evidence). No differences between strategies were detected by subgroup analysis (p=0.25). Up to 5 years after treatment, slightly fewer cases of PTS occurred in those receiving thrombolysis, 50% vs 53% with standard anticoagulation (RR 0.78; 95% CI, 0.66-0.93; 1393 participants, 6 studies; moderate-certainty evidence). This was still observed at late follow-up (beyond 5 years) in 2 studies (RR 0.56; 95% CI, 0.43-0.73; 211 participants; moderate-certainty evidence). Using subgroup analysis to investigate if the level of DVT (iliofemoral, femoropopliteal, or non-specified) had an effect on the incidence of PTS, no benefit of thrombolysis was observed regardless of the level of DVT (6 studies; test for subgroup differences: p=0.29). Systemic thrombolysis and CDT had similar levels of effectiveness. Studies of CDT included 4 trials in femoral and iliofemoral DVT, and the results are consistent with those from trials of systemic thrombolysis in DVT at other levels of occlusion.

### Recommendation 18

In selected patients with symptomatic acute iliofemoral DVT (especially with low risk of bleeding), we recommend the use of early thrombus removal strategies. Class II, level B^147-153^

### Recommendation 19

In patients with DVT involving the femoral, popliteal, and/or calf veins,

early thrombus removal is not recommended. Class II, level C^147-153^

### Recommendation 20

In patients with acute lower extremity DVT who have undergone thrombus removal, anticoagulation should have the same intensity and duration as that indicated for patients who have not undergone a thrombus removal procedure. Class I, level B^147-153^

## COVID-19 and VTE

### COVID-19-related VTE

#### Epidemiology

An increased risk of thromboembolic events in patients infected with SARS-CoV-2 has been demonstrated by several studies,^156-160^ as well as higher mortality when these events are associated, making it necessary to suspect them especially in patients with moderate-to-severe infection admitted to wards or intensive care units (ICUs).

Thrombotic events in the venous system and their consequences, which include DVT and PE, are generally more common in hospitalized patients, especially in ICUs,^161^ with an incidence of VTE in hospitalized patients without thromboprophylaxis of 14.9%.^161,162^

According to a meta-analysis published in the Chest Journal in 2021, in patients with COVID-19 requiring hospitalization, the rate of VTE was 17%, PE was 7.1%, and DVT was 12.1%.^163^ In another recent meta-analysis, the prevalence of DVT and PE in hospitalized patients diagnosed with COVID-19 was even higher (14.8% and 16.5%, respectively).^164^

### Diagnosis

#### Laboratory testing

DD levels are elevated in most patients with SARS-CoV-2 infection even without VTE, but some studies have shown even higher DD levels in patients with COVID-19 associated with DVT and PE.^165-167^

Demelo-Rodríguez et al.^167^ reported that a DD cutoff point of 1.57 (μg/mL) has a sensitivity of 95.7%, specificity of 29.3%, positive predictive value of 19%, and negative predictive value of 97.5% for the diagnosis of asymptomatic DVT in patients with COVID-19. Elevated DD was also considered as a predictor of severity.^168,169^

### Treatment

The principles of VTE treatment in patients with COVID-19 do not differ from those in patients with non-COVID-19 VTE.^170^ The use of parenteral anticoagulants is recommended as initial therapy (UFH and LMWH), especially in hospitalized patients, with the possibility of transitioning to oral anticoagulants (DOACs or VKAs) after clinical stabilization.^171^ DOACs have been the therapy of choice in the transition from parenteral to oral regimens because they have lower bleeding rates than warfarin. Factor Xa inhibitors, such as rivaroxaban and apixaban, have been the most widely used to treat VTE in patients with COVID-19 after hospital discharge and stabilization of the patient’s clinical condition. Treatment duration will depend on whether or not risk factors persist, in addition to concomitant diseases that require full-dose anticoagulation, but it should be considered for at least 3 months.^172^

### Anticoagulation in patients with COVID-19 without evidence of VTE

This topic has raised many questions and discrepant studies in the literature. There is no doubt that patients with COVID-19 with evidence of VTE should receive a standard therapeutic anticoagulation regimen, and the same should be administered to patients without COVID-19. The crux of the issue is what type of anticoagulant regimen should be administered to patients with COVID-19 without evidence of thrombotic events. Two studies have recently evaluated therapeutic-dose and prophylactic-dose anticoagulation regimens in 2 types of patients: critically ill patients requiring orotracheal intubation, and hospitalized patients with moderate COVID-19 not requiring supplemental oxygen therapy. The first study evaluated the types of anticoagulation in critically ill patients, already on orotracheal intubation, admitted to the ICU. A total of 1098 patients were evaluated: one group of patients receiving UFH or enoxaparin at a therapeutic dose, and the other group of patients receiving enoxaparin or UFH at a prophylactic dose. The results of the study showed that survival to hospital discharge was 62.7% in patients on a therapeutic-dose regimen vs 64.5% in patients on a prophylactic-dose regimen, with no statistical difference between the groups. The rate of major bleeding, however, was higher with therapeutic-dose anticoagulation than with prophylactic-dose anticoagulation (3.8% vs 2.3%). The authors concluded that there was no difference in mortality between critically ill patients receiving therapeutic-dose and prophylactic-dose anticoagulation, but there was a higher rate of bleeding among patients receiving full-dose anticoagulation.^173^ The INSPIRATION study, which evaluated 600 patients admitted to the ICU, randomized to treatment with full-dose vs prophylactic-dose anticoagulation, found no benefit with therapeutic-dose anticoagulation in preventing mortality, thromboembolic events, or need for extracorporeal membrane oxygenation (HR 1.06; 95% CI, 0.83-1.36).^174^

Another study aimed to evaluate therapeutic-dose vs prophylactic-dose anticoagulation with enoxaparin or UFH in hospitalized patients with moderate COVID-19, without target organ damage or failure and no need for supplemental oxygen therapy. Among 2219 patients in the final analysis, the probability that therapeutic-dose anticoagulation increased organ support-free days, as compared with usual-care thromboprophylaxis, was 98.6%. The final probability of the superiority of therapeutic-dose anticoagulation over usual-care thromboprophylaxis was 97.3% in patients with high DD levels, 92.9% in patients with low DD levels, and 97.3% in patients with unknown DD levels. Major bleeding occurred in 1.9% of patients receiving therapeutic-dose anticoagulation and in 0.9% of those receiving thromboprophylaxis. The authors concluded that full-dose anticoagulation with enoxaparin or UFH in patients with moderate COVID-19 increased the probability of survival to hospital discharge with a lower incidence of multisystem organ failure, that is, renal, cardiovascular, and respiratory failure.^172,173^

Likewise, the HEP-COVID trial evaluated 253 patients with COVID-19 and elevated DD levels, randomized to therapeutic-dose or prophylactic-dose anticoagulants. The primary outcomes (death, VTE, and arterial thrombosis) were higher in the group receiving prophylactic-dose anticoagulation (42%) than in the group receiving full-dose anticoagulation (29%) (RR 0.68; 95% CI, 0.49-0.96; p=0.03).^175^ This benefit was only observed in patients on medical wards. Patients admitted to the ICU did not benefit from full-dose anticoagulation.

Overall, based on the literature, in critically ill patients admitted to the ICU with COVID-19, the use of prophylactic-dose anticoagulation is recommended (SC enoxaparin 40 mg once daily). For hospitalized patients with COVID-19 on medical wards, therapeutic-dose anticoagulation is recommended (SC rivaroxaban 20 mg/day or SC enoxaparin 1 mg/kg/dose, every 12 hours). In turn, patients hospitalized for other clinical conditions, in whom COVID-19 is discovered incidentally, prophylactic-dose anticoagulation is sufficient, being recommended in the general literature.

A potential benefit of maintaining anticoagulation at prophylactic doses after hospital discharge of patients with COVID-19, at high risk of VTE, was demonstrated in the MICHELLE study,^176^ which evaluated 320 hospitalized patients with COVID-19 at high risk of developing VTE but with no documented evidence of VTE at the time of discharge, randomized to receive rivaroxaban 10 mg or no anticoagulation for 35 days after hospital discharge. The incidence of complications (VTE, arterial thromboembolism, or cardiovascular events) was higher in patients receiving no anticoagulation (9%) than in those receiving prophylactic-dose rivaroxaban (3%) (RR 0.33; 95% CI, 0.12-0.90). Despite the robust data, this is a small cohort. Therefore, there is still no consensus in the literature on the routine recommendation of prophylactic-dose anticoagulation for patients at high risk of VTE discharged after hospitalization for COVID-19, and a case-by-case analysis is recommended, weighing the risks and benefits for each patient, pending new studies in the literature.

Another study, which followed 4906 hospitalized patients with COVID-19 who were discharged to outpatient care, showed an incidence of VTE of 1.6% post-discharge, with pharmacological prophylaxis for VTE being used only in 13% of the sample. The incidence of VTE was lower in patients not receiving anticoagulants than in those receiving pharmacological prophylaxis for VTE in this study sample. The rate of major bleeding with anticoagulation was 1.7%, higher than the risk of VTE.^177^

Most patients with COVID-19 are treated on an outpatient basis, not requiring hospital admission. Pharmacological prophylaxis for VTE is not indicated for these patients unless they have other associated factors that require anticoagulant use, including current documented VTE, atrial fibrillation, active cancer, and inherited thrombophilia posing a high risk of VTE, such as antithrombin deficiency and protein C deficiency, among others. Each patient should be evaluated individually, weighing the risks and benefits of using anticoagulants as pharmacological prophylaxis for VTE in patients with COVID-19 treated on an outpatient basis not requiring hospitalization.

In summary, VTE treatment in patients with COVID-19 follows principles similar to those of treatment of VTE unrelated to COVID-19. The use of parenteral anticoagulants is recommended as initial therapy (UFH and LMWH) in hospitalized patients, with the possibility of transitioning to oral anticoagulants (DOACs or VKAs) after clinical stabilization. DOACs, such as rivaroxaban and apixaban, are preferred due to reduced bleeding rates compared with warfarin.

Therefore, the decision to use post-discharge VTE pharmacological prophylaxis should be personalized, considering the risk-benefit ratio. In outpatients, prophylaxis is not systematically recommended, except in the presence of risk factors and taking into account the likelihood of bleeding, as well as the patient’s additional clinical conditions.

### Recommendation 21

In acute thromboembolic events in patients with COVID-19, we recommend that the anticoagulation decision should follow the same principles as for events that occur without the concomitant viral infection. Class II, level A^172-175^

### Recommendation 22

In outpatients with COVID-19 and in the absence of other clinical conditions that indicate the use of anticoagulants, the administration of these medications is not recommended. Class I, level B^172-175^

### Recommendation 23

In patients hospitalized for COVID-19, we recommend categorizing the risk of post-discharge venous thromboembolic events before deciding to use outpatient anticoagulation. Class II, level A^177,178^
