# Supplementary material for: Guidelines on deep vein thrombosis of the Brazilian Society of Angiology and Vascular Surgery
Source: J Vasc Bras. 2024 Sep 3;23:e20230107. doi: 10.1590/1677-5449.202301072 (PMC11404781; doi:10.1590/1677-5449.202301072)
Supplement: Translation

# Translation: Diretrizes sobre trombose venosa profunda da Sociedade Brasileira de Angiologia e Cirurgia Vascular

**DOI:** 10.1590/1677-5449.202301071

**Authors:** Marcone Lima Sobreira, Marcos Áreas Marques, Adilson Ferraz Paschoa, Alcides José Araújo Ribeiro, Ivan Benaduce Casella, Marcelo Calil Burihan, Marcelo Fernando Matielo, Rafael de Athayde Soares, Walter Junior Boin de Araujo, Edwaldo Edner Joviliano, Julio Cesar Peclat de Oliveira

**Affiliations:** 1 Universidade Estadual Paulista “Júlio de Mesquita Filho” – UNESP, Departamento de Cirurgia e Ortopedia, São Paulo, SP, Brasil.; 2 Universidade do Estado do Rio de Janeiro – UERJ, Departamento de Medicina Interna, Rio de Janeiro, RJ, Brasil.; 3 Associação Portuguesa de Beneficência de São Paulo, Centro de Cirurgia Vascular Integrada, Real e Benemérita, São Paulo, SP, Brasil.; 4 Hospital de Base do Distrito Federal, Serviço de Cirurgia Vascular, Brasília, DF, Brasil.; 5 Universidade de São Paulo – USP, Departamento de Cirurgia, São Paulo, SP, Brasil.; 6 Hospital Santa Marcelina, Departamento de Cirurgia Vascular, Endovascular e Ecografia Vascular, São Paulo, SP, Brasil.; 7 Hospital do Servidor Público Estadual de São Paulo, Departamento de Cirurgia Vascular e Endovascular, São Paulo, SP, Brasil.; 8 Universidade Federal do Paraná – UFPR, Hospital das Clínicas – HC, Curitiba, PR, Brasil.; 9 Sociedade Brasileira de Angiologia e de Cirurgia Vascular – SBACV-PR, Curitiba, PR, Brasil.; 10 Universidade de São Paulo – USP, Faculdade de Medicina de Ribeirão Preto – FMRP, Ribeirão Preto, SP, Brasil.; 11 Sociedade Brasileira de Angiologia e de Cirurgia Vascular – SBACV-SP, São Paulo, SP, Brasil.; 12 Universidade Federal do Estado do Rio de Janeiro – UNIRIO, Programa de Pós-graduação de Cirurgia Vascular, Rio de Janeiro, RJ, Brasil.

**Keywords:** trombose venosa profunda, tromboembolia venosa, ultrassonografia Doppler duplex, anticoagulantes, fibrinólise, filtros de veia cava

## Abstract

Trombose venosa profunda é uma das principais causas de morbidade hospitalar e ambulatorial, seja em pacientes clínicos, seja em pacientes cirúrgicos, impactando significativamente nas estatísticas de mortalidade, exigindo um diagnóstico rápido para que se institua de forma imediata o tratamento. O presente documento foi preparado e revisado por onze especialistas certificados pela Sociedade Brasileira de Angiologia e Cirurgia Vascular, que buscaram nas principais bases de dados as melhores evidências referentes à abordagem diagnóstica (exame físico, exames de imagem) e terapêutica (heparina, cumarínicos, anticoagulantes orais de ação direita, fibrinolíticos) da doença.

## INTRODUÇÃO

A trombose venosa profunda (TVP) caracteriza-se pela formação de trombos dentro de veias profundas, com obstrução parcial ou oclusão, sendo mais comum nos membros inferiores – em 80-95% dos casos^1,2^ . As principais complicações decorrentes dessa doença são insuficiência venosa crônica/síndrome pós-trombótica (SPT) (edema e/ou dor em membros inferiores, mudança na pigmentação, ulcerações na pele) e embolia pulmonar (EP). Esta última tem alta importância clínica, por apresentar alto índice de mortalidade^1,2^ .

Aproximadamente 5 a 15% de indivíduos não tratados da TVP podem morrer de EP^3^ . TVP ou EP podem ocorrer em 2/1.000 indivíduos a cada ano, com uma taxa de recorrência de até 25%^3^ . A rápida adoção de estratégias diagnósticas e terapêuticas é crucial para evitar essas complicações.

## FATORES DE RISCO

Os principais fatores diretamente ligados à gênese dos trombos são estase sanguínea, lesão endotelial e estados de hipercoagulabilidade^2^ . Portanto, idade avançada, câncer, procedimentos cirúrgicos, imobilização, uso de estrogênio, ciclo gravídico-puerperal, distúrbios de hipercoagulabilidade hereditários ou adquiridos, constituem-se como fatores de risco para TVP^3,4^ . A sua incidência aumenta proporcionalmente com a idade, sugerindo que esse seja o fator de risco mais determinante para um primeiro evento trombótico^5^ .

Para efeitos didáticos, os fatores de risco podem ser classificados como^6^ :

- Hereditários/idiopáticos: resistência à proteína C ativada (principalmente fator V Leiden); mutação do gene da protrombina G20210A; deficiência de antitrombina; deficiência de proteína C; deficiência de proteína S; aumento do fator VIII; aumento do fibrinogênio;
- Adquiridos/provocados: síndrome do anticorpo antifosfolipídio (SAF); câncer; hemoglobinúria paroxística noturna; idade > 65 anos; sobrepeso e obesidade; gravidez e puerpério; doenças mieloproliferativas (policitemia vera; trombocitemia essencial etc.); síndrome nefrótica; hiperviscosidade (macroglobulinemia de Waldenström; mieloma múltiplo); doença de Behçet; trauma; cirurgias; imobilização; terapia estrogênica.

## CLASSIFICAÇÃO

A TVP nos membros inferiores é dividida, segundo sua localização:

- Proximal: quando acomete veia ilíaca e/ou femoral e/ou poplítea;
- Distal: quando acomete as veias localizadas abaixo da veia poplítea.

O risco de EP e a magnitude da SPT decorrente da TVP proximal são maiores. Entretanto, existe risco de progressão da TVP distal para segmentos proximais de até 20%, o que faz com que o seu diagnóstico e tratamento sejam similares ao da TVP proximal^7^ . Portanto, a classificação do tipo de TVP suspeita é importante para guiar as estratégias de tratamento.

## DIAGNÓSTICO

### Exame físico

Os principais sinais e sintomas, quando presentes, são: dor, edema, eritema, cianose, dilatação do sistema venoso superficial, aumento de temperatura e empastamento muscular^7^ . Os principais fatores relacionados ao surgimento da TVP, associados ao quadro de dor e edema, podem ser agrupados em modelos de predição clínica^8^ .

Nenhuma avaliação clínica feita de forma isolada é suficiente para diagnosticar ou descartar a TVP^1^ , pois os achados clínicos se relacionam com a doença em apenas 50% dos casos^2^ . A literatura existente recomenda a anamnese e o exame físico, combinados com a realização de testes laboratoriais e exames de imagem^1,8,9^ . O sistema de predição clínica de TVP mais bem estudado é o escore de Wells (EW)^9,10^ .

### Escore de Wells (EW)

É um modelo de predição clínica, baseado em sinais e sintomas, fatores de risco e diagnósticos alternativos, estimando a probabilidade pré-teste para TVP (Tabela 1). Esse escore tem se mostrado útil na abordagem inicial do paciente com suspeita de TVP^8,9,12^ . Em sua primeira versão, categoriza os pacientes com probabilidade baixa, moderada ou alta de TVP, com uma prevalência de 5% (IC95% 4-8), 17% (IC95% 13-23) e 53% (IC95% 44-61), respectivamente^10^ .

**Tabela 1:** Terapia trombolítica associada à anticoagulação em comparação com anticoagulação isolada em pacientes com TVP proximal extensa.

**Desfecho**	**Risco relativo (IC 95%)**	**Risco com anticoagulação**	**Diferença de risco com terapia trombolítica + anticoagulação**
Mortalidade	0,77 (0,26-2,28)	9 por 1.000	2 eventos a menos por 1.000
Síndrome pós-trombótica	0,71 (0,60-0,085)	641 por 1.000	186 menos eventos de SPT/1.000
Sangramento maior	1,85 (1,45-2,44)	36 por 1.000	31 eventos a mais por 1.000

TVP **=** Trombose venosa profunda. Adaptada de American Society of Hematology 2020 guidelines^11^ .

O EW deve ser usado em combinação com métodos diagnósticos adicionais, como o eco-Doppler colorido (EDC) associado à compressão de todo trajeto venoso troncular do membro inferior (pacientes com EW de alta probabilidade) e a mensuração do D-dímero (DD) (pacientes com EW de baixa probabilidade)^1,9^ . A combinação EDC negativa seguida de DD negativo permite descartar com segurança a hipótese da TVP^8^ . Metanálise elaborada por Geersing et al.^8^ define que, com EW ≤1 e DD negativo, a probabilidade de TVP é menor que 2%, podendo ser excluída com segurança em diferentes grupos de pacientes, com exceção da presença de câncer e TVP recorrente. O EW apresenta melhor resultado na avaliação de pacientes jovens sem comorbidades ou história prévia de tromboembolismo venoso (TEV) do que em outros pacientes^13^ . Para TVP recorrente é recomendado usar o EW modificado (que inclui pontuação extra para a história prévia de TVP)^8^ .

### Exame laboratorial

#### DD no diagnóstico e acompanhamento do TEV

O DD é um marcador molecular que resulta da dissolução da fibrina. Costuma estar elevado em eventos trombóticos, mas também pode estar elevado em outras situações, como gestação, câncer, trauma, infecção e outros processos pró-inflamatórios^1^ . A sensibilidade desse marcador varia de 94 a 100%, de modo que a ausência de elevação sérica de DD afasta um diagnóstico de TVP em 97 a 98% dos casos. Seus valores situam-se em grupos: negativo (<350 ng/mL), intermediário (351-500 ng/mL) e positivo (>500 ng/mL)^13,14^ .

Quanto mais avançada a idade do paciente, maior o valor do DD, o que diminui e especificidade do teste. Uma revisão sistemática publicada em 2014 demonstrou a necessidade de se ajustar o valor referência do DD com a idade (idade multiplicada por 10 mcg/L) em pacientes mais velhos que 50 anos, e isso aumentou a especificidade sem alterar a sensibilidade do teste laboratorial^14^ . A gestação também aumenta o valor do DD, o que pode reduzir a especificidade do teste, caso seja necessária sua avaliação em um eventual diagnóstico de TVP^14,15^ . Em um pequeno estudo com mulheres assintomáticas, nenhuma das gestantes apresentava um valor de DD menor 0,50 mg/L no terceiro trimestre. Os autores concluíram que um valor maior deveria ser usado com referência no primeiro trimestre (0,75 mg/L), no segundo trimestre (1 mg/L) e no terceiro trimestre (1,5 mg/L)^16^ .

### Recomendação 1

Quando combinado com um resultado negativo no teste DD, é possível excluir a TVP em pacientes que apresentam uma pontuação de baixa probabilidade no EW. Classe I, nível A9

#### DD e o risco de TEV

Em pacientes internados com doenças críticas, a elevação do DD está associada a um risco aumentado de TEV, independentemente da idade, sexo, raça, índice de massa corpórea e doença clínica^17^ . O estudo Magellan avaliou 7.581 pacientes hospitalizados com doenças críticas e observou que a incidência de TEV foi 3,5 vezes maior entre os pacientes com concentração sérica de DD maior ou igual a duas vezes o valor normal (OR 2,29; IC95% 1,75-2,98)^17^ . Na população em geral, um DD elevado esteve associado a maior risco de um primeiro episódio de TEV, em um estudo que avaliou 923 pacientes durante 8 anos^18^ .

Entre portadores de câncer, os níveis de DD são consideravelmente elevados nos pacientes que apresentam o primeiro episódio de TEV^19^ . Em um estudo que avaliou 821 pacientes com câncer e concentração de DD duas vezes maior que o valor normal, a incidência de TEV foi 1,3 vezes tanto em uma análise univariada (IC95% 1,1-1.5; p < 0.001) quanto multivariada (HR 1,3; IC95% 1,2-1,6; p < 0.001)^20^ . A associação entre níveis elevados de DD e a ocorrência do primeiro episódio de TEV foi validada em vários estudos, incluindo câncer colorretal, pulmão e câncer ginecológico^21^ .

#### DD e recorrência de TEV

Em relação à recorrência de TEV e o valor do DD, há uma emblemática revisão sistemática na literatura, envolvendo 1.888 pacientes com um primeiro episódio de TEV não provocada, que demonstrou que os pacientes que mantinham níveis de DD elevados após a descontinuação da terapia anticoagulante apresentaram incidência anual de TEV de 8,9%, comparada com a incidência de 3,5% de TEV em pacientes com níveis normais de DD^22^ . Outra metanálise realizada com a mesma população demonstrou incidência anual de TEV recorrente de 8,8% em pacientes com níveis elevados de DD, contra 3,7% em pacientes com níveis normais de DD^23^ . Estudo publicado recentemente demonstrou uma associação entre níveis elevados de DD com o risco de recorrência de TEV entre 3 semanas (HR 1,4; 1,06-1,84) até 15 meses (HR 1,26;1,01-1,57) após a descontinuidade da terapêutica com anticoagulantes^24^ . Apesar de não existirem diretrizes internacionais nem nacionais orientando manutenção ou suspensão dos anticoagulantes com base no valor do DD, é recomendada que se faça a dosagem deste após 1 mês da suspensão do anticoagulante. Caso o mesmo se mantenha elevado, é preciso considerar o resultado desses dois artigos científicos, que demonstram incidência quase dobrada de recidiva de TEV em pacientes com níveis elevados de DD quando comparados aos pacientes com níveis normais.

### Recomendação 2

Dosagem sérica do DD persistentemente elevado após a parada da anticoagulação pode indicar risco de recorrência, porém não prediz maior ou menor risco de recorrência de evento trombótico. Classe I, nível A23.

#### DD e COVID-19

A discussão a respeito do DD e o risco de TEV ganhou atenção especial devido à pandemia da COVID-19, já que foram observados valores altos deste em pacientes acometidos pela infecção, principalmente devido ao seu caráter pró-inflamatório e acometimento vascular e endotelial. Um estudo randomizado, o HEP-COVID, recrutou pacientes infectados pela COVID-19 com valores de DD quatro vezes maiores do que o valor de referência e randomizou os pacientes para tratamento com heparina de baixo peso molecular (HBPM) na dose profilática *versus* dose terapêutica. O grupo de pacientes infectados pela COVID-19 submetidos ao tratamento com anticoagulação plena apresentou incidência de TEV de 10,9%, comparados aos 29% no grupo de pacientes submetidos à anticoagulação profilática (RR 0,37; IC95% 0,21-0,66; p <.001).

Além disso, o grupo de pacientes submetidos à anticoagulação plena apresentou menores taxas de mortalidade (19,4%) em relação aos pacientes submetidos à anticoagulação profilática (25%), (RR 0,68; IC95% 0,49-0,96; p = 0,03)^25^ . Alguns estudos e metanálises evidenciaram uma associação entre valores altos de DD e mortalidade por COVID-19, cujo mecanismo fisiopatológico está associado a um estado secundário hiperativo de fibrinólise, ou consumo exacerbado de fibrina^26^ . Uma metanálise publicada em 2021 encontrou associação entre o nível elevado de DD e mortalidade em pacientes internados com infecção por COVID-19, com menor probabilidade de alta hospitalar, maior progressão para intubação orotraqueal e ventilação mecânica, mostrando que níveis maiores de DD na admissão hospitalar desses pacientes indicariam um maior estado pró-inflamatório, com uma cascata de interleucinas em ativação máxima e estado de hiperfibrinólise^27^ . Outra metanálise, de 2021, avaliou 10.491 pacientes infectados por COVID-19 e evidenciou uma forte associação entre níveis elevados de DD e mau prognóstico na evolução da doença (OR 3,39; IC95% 2,66-4,33; p < 0.00001)^28^ . Em suma, os níveis elevados de DD na admissão dos pacientes infectados pela COVID-19 estão associados a um mau prognóstico, com maior risco de TEV, evolução para ventilação mecânica e óbito. Não existe um limite referencial de corte para o valor de DD; entretanto, alguns estudos sugerem que o valor acima de 2,0 μg/mL na admissão dos pacientes poderia ser um fator preditor de mau prognóstico nesses pacientes que necessitem internação^29^ .

Em resumo, a dosagem sérica do DD em pacientes com COVID-19 desempenha um papel importante na avaliação do estado de coagulação, inflamação e risco de complicações, como trombose e gravidade da doença. No entanto, a interpretação dos resultados deve ser feita considerando o contexto clínico individual e outros marcadores clínicos, e a tomada de decisões clínicas deve ser orientada por profissionais de saúde experientes.

#### DD e a SPT

A SPT é uma das mais nefastas complicações da TVP de membros inferiores devido à sua morbidade, com impacto na qualidade de vida e nas atividades socioeconômicas do paciente. Apesar de ser um preditor de processo inflamatório e atividade trombótica, os valores de DD no seguimento de pacientes com TEV não estão relacionados ao desenvolvimento de SPT, como foi documentado em diversos trabalhos na literatura^30,31^ .

Trombo residual detectado ao EDC, recanalização venosa parcial ou ausente, tipo de anticoagulante administrado durante o tratamento da TVP, refluxo na veia poplítea e idade avançada permanecem como principais fatores de risco determinantes para a SPT.

Em resumo, o DD desempenha um papel importante no diagnóstico, acompanhamento e avaliação do risco de TEV em diversos contextos clínicos. Os níveis de DD podem ser influenciados por diferentes fatores, como idade e condições médicas subjacentes, e seu valor prognóstico varia de acordo com o cenário clínico. Portanto, a interpretação adequada dos resultados do DD é fundamental para orientar o diagnóstico e o tratamento de pacientes com TEV e outras condições médicas.

### Exame de imagem

#### EDC

O EDC venoso é o método complementar mais frequentemente utilizado para o diagnóstico de TVP em pacientes sintomáticos. Apresenta menor acurácia em veias distais, em veias de membros superiores e em pacientes assintomáticos^7,9,32,33^ . Atualmente, é o exame de escolha para o diagnóstico de TVP^7,9,34^ , com sensibilidade de 96% e especificidade de 98 a 100%^35^ , em substituição à venografia^7,32,35,36^ .

Utiliza-se a ultrassonografia em tempo real para avaliar a ausência ou presença de compressibilidade das veias e a ecogenicidade intraluminal. O EDC avalia a anatomia e as características do fluxo venoso, combinando imagem em tempo real e a análise espectral^7,9,37^ . A indicação de exame de todo o membro é favorecida, em relação ao exame segmentar ou POCUS (do inglês, *point of care ultrasound*), embora ainda seja um assunto polêmico em certas instituições^38^ .

No paciente com alta probabilidade de acordo com o EW, EDC negativo e DD positivo, o EDC deverá ser repetido em 3 a 7 dias. Nos casos de TVP recorrente ipsilateral, os critérios utilizados para o diagnóstico pelo EDC são: aumento do diâmetro do mesmo segmento acometido ≥ 4 mm, aumento de 9 cm de extensão do trombo, ou em segmento venoso distinto do acometido previamente^36^ . O EDC pode distinguir entre processos patológicos não vasculares, como adenopatia inguinal, cisto de Baker, abscesso e hematomas, entre outros.

No seguimento dos pacientes diagnosticados com TVP aguda, o consenso da *Society of Radiologists in Ultrasound* de 2018 recomenda a utilização do termo “alterações pós-trombóticas crônicas” em vez de “TVP crônica”, a fim de evitar confusão com a TVP em curso. Também recomenda cautela no emprego do termo “TVP subaguda”, devido à dificuldade na sua caracterização^39,40^ .

O EDC desempenha um papel fundamental no diagnóstico de TVP, oferecendo alta sensibilidade e especificidade. Além disso, é útil no seguimento de pacientes com TVP aguda, permitindo a diferenciação de outras condições não vasculares. A terminologia recomendada para descrever alterações pós-trombóticas crônicas visa evitar confusões na comunicação médica.

A EDC e a dosagem sérica do DD é frequentemente usada para o diagnóstico e confirmação da TVP. A sensibilidade e especificidade dessa combinação podem variar dependendo das circunstâncias clínicas, dos critérios de diagnóstico e dos valores de corte utilizados para o DD. No entanto, em geral:

1. Sensibilidade: A combinação de EDC e dosagem de DD é conhecida por apresentar alta sensibilidade. Isso significa que a maioria dos casos de TVP é identificada corretamente por essa abordagem. A sensibilidade pode variar, mas muitos estudos relatam sensibilidades superiores a 90% para o diagnóstico de TVP;
2. Especificidade: A especificidade da combinação EDC e DD é geralmente alta, mas pode ser menor do que a sensibilidade. Isso ocorre porque a elevação dos níveis de DD pode ocorrer em várias condições médicas além da TVP, como infecções, inflamações e doenças crônicas. Portanto, a especificidade pode variar, mas é comum encontrar valores acima de 80% para a combinação.

É importante ressaltar que os valores de corte para o DD podem afetar a sensibilidade e especificidade da abordagem. Valores de corte mais baixos aumentarão a sensibilidade, mas podem diminuir a especificidade, enquanto valores de corte mais altos aumentarão a especificidade, mas podem diminuir a sensibilidade.

### Recomendação 3

EW de moderada ou baixa probabilidade associado com dosagem sérica de DD normal podem prescindir da necessidade de realização de EDC, para exclusão diagnóstica. Classe II nível C23

### Recomendação 4

EW de alta probabilidade associado com dosagem sérica de DD elevada sugerem necessidade de realização de EDC, para confirmação diagnóstica. Classe II nível C23.

#### Venografia/flebografia

A flebografia é o exame considerado padrão-ouro para o diagnóstico de TVP, reservado, atualmente, apenas quando os outros testes são incapazes de definir o diagnóstico. Porém, devido a várias limitações (custo, reações adversas ao contraste, ser desconfortável para o paciente, contraindicado a pacientes com insuficiência renal e usar radiação), não é o exame de rotina utilizado na suspeita de TVP. Tem acurácia limitada nos quadros de TVP recorrente^1^ . A flebografia pode ser útil nos casos em que a EDC sugere a presença de obstrução proximal ao demonstrar perda da fasicidade do fluxo, mas não consegue visualizar o trombo nas veias ilíacas^41^ .

#### Angiotomografia computadorizada (ATC)

Como a sensibilidade e especificidade da ATC são similares à do EDC, não há evidência suficiente para recomendá-la como modalidade diagnóstica inicial para TVP^36^ . Em metanálise realizada por Thomas et al.^42^ , a sensibilidade encontrada para ATC foi de 96% (IC95% 93-98), e especificidade de 95% (IC95% 94-97) para diagnóstico de TVP proximal em pacientes com suspeita de EP. Pode ser útil para pacientes com suspeita de TVP, para os quais o EDC não pode ser aplicado devido a limitações técnicas e suspeita de anomalia venosa^36^ .

O uso da ATC tem o benefício de avaliar as veias pélvicas e a veia cava inferior (VCI), cuja avaliação é limitada no EDC, além de detectar outras etiologias de edema de membros inferiores, como neoplasia pélvica ou compressão extrínseca por outras estruturas vasculares, incluindo a síndrome de compressão da veia ilíaca comum esquerda (síndrome de May-Thurner)^43^ . Peterson et al.^44^ demonstraram que, embora a sensibilidade seja alta, em 93%, produzindo um valor preditivo negativo de 97%, a capacidade da ATC venosa para diagnosticar TVP tem uma especificidade de 71%, dando um valor preditivo positivo de apenas 53%; outros pesquisadores mostraram uma taxa de falso positivo de 50% para ATC venosa para TVP pélvica.

#### Angiorressonância magnética (ARM)

A ARM pode ser utilizada para o diagnóstico de TVP em casos onde o EDC oferece resultados inconclusivos^36,45^ . Apresenta acurácia similar ao EDC no diagnóstico da TVP do segmento ilíaco-caval^42,46^ . A ARM com imagem direta do trombo, baseada nas propriedades paramagnéticas da metaemoglobina, pode ser o método de escolha para suspeita de recorrência aguda de TVP, distinguindo um evento novo de um antigo^36^ .

Estudos comparando a ARM com a flebografia encontraram sensibilidade e especificidade da ARM de até 100% para o diagnóstico de TVP fêmoro-poplítea, porém a acurácia foi menor para trombos distais. Na avaliação de TVP proximal, a ARM tem acurácia semelhante ao EDC e à flebografia, com o benefício de altas taxas de detecção da extensão proximal do trombo e trombos pélvicos isolados^47^ .

Embora a ARM demonstre excelente precisão para a detecção de TVP aguda, ela apresenta várias desvantagens. O exame requer um paciente imóvel para tempos de imagem longos, e não pode ser usado em pacientes com dispositivos ou clipes metálicos. O contraste utilizado, gadolínio, pode ser tóxico em pacientes com disfunção renal, e a necessidade de exames frequentes pode produzir problemas de utilização em instituições maiores. Finalmente, a qualidade da ARM depende da experiência técnica da instituição^47^ .

Em resumo, a venografia/flebografia é o padrão-ouro, mas é reservada para casos em que outros testes não fornecem diagnóstico. A ATC tem sensibilidade e especificidade semelhantes à EDC, enquanto a ARM é útil em casos de EDC inconclusivo e pode distinguir eventos novos de antigos em recorrências. A escolha da modalidade depende das circunstâncias clínicas e das limitações técnicas e de segurança do paciente. Pode-se optar pela realização de ATC ou ARM (em comparação com a venografia) quando não for possível a realização do EDC.

## TRATAMENTO

### Anticoagulação no evento agudo (pacientes convencionais)

O tratamento da TVP é um desafio e tem como base o uso de anticoagulantes, a fim de evitar a progressão do trombo enquanto a ativação dos mecanismos fibrinolíticos primários promove a sua dissolução^48-51^ . A escolha do anticoagulante inicial inclui: disponibilidade; familiaridade de uso pela equipe médica; características farmacocinéticas e dinâmicas; comorbidades do paciente; facilidade de reversão de seu efeito; e até preferência do paciente e seu custo^52^ . Hoje, temos disponíveis para uso no Brasil a heparina não fracionada (HNF), as HBPM, a fondaparinux, os antagonistas da vitamina K (AVKs) e os DOACs (do inglês, *direct oral anticoagulants*).

Não há anticoagulante totalmente seguro no que diz respeito a hemorragias. Assim, na prática clínica, o risco de hemorragia deve ser sempre avaliado no tratamento do TEV. Os diversos anticoagulantes podem apresentar diferentes riscos para hemorragias, dependendo: da intensidade do tratamento; do uso concomitante de outros anticoagulantes, drogas trombolíticas ou antiplaquetárias; características do paciente; e comorbidades^53^ .

### Recomendação 5

Em pacientes com TVP proximal, a abordagem preferencial é geralmente o uso de terapia anticoagulante como tratamento primário, em vez de combinar com terapia trombolítica. Classe II nível C53-55.

#### Duração da anticoagulação

O objetivo em prolongar a duração do tratamento é prevenir a recorrência de TVP. O risco é menor se a TVP ocorre na presença de fatores de risco reversíveis, como por exemplo cirurgias; e maior, se a TVP for idiopática ou associada ao câncer. Pacientes com EP sintomática também têm maior risco de recorrência do que aqueles com apenas TVP. O risco também é maior na presença de outros complicadores, tais como trombofilias hereditárias em homozigose, síndrome do anticorpo fosfolipídio ou combinação de trombofilias^53^ .

#### Anticoagulação inicial para TVP aguda de membros inferiores

Para pacientes com alta suspeita clínica de TVP, recomenda-se iniciar o tratamento com anticoagulantes enquanto se aguarda a confirmação do diagnóstico, desde que não haja contraindicação^54^ . Na maioria dos pacientes com TVP proximal, sugere-se terapia anticoagulante isolada em vez de associação com terapia trombolítica (recomendação condicional, baixa certeza), conforme demonstrado na Tabela 1.

#### Observações

Pacientes com membro ameaçado por TVP podem requerer trombólise. Em pacientes com TEV (Tabela 2), sugere-se o uso de DOACs ao invés de AVKs (recomendação condicional, certeza moderada).

**Tabela 2:** DOACs comparados com AVK para TEV.

**Desfecho**	**Risco relativo (IC95%)**	**Risco com antivitamina K**	**Diferença de risco com DOAC**
Mortalidade	0,99 (0,85-1,15)	39 por 1.000	Evento nulo
Embolia pulmonar	0,97 (0,77-1,23)	20 por 1.000	1 evento de EP a menos por 1.000
Trombose venosa profunda	0,80 (0,59-1,09)	26 por 1.000	5 eventos de TVP a menos por 1.000
Sangramento maior	0,63 (0,47-0,84)	17 por 1.000	6 eventos de sangramento a menos por 1.000

DOAC = do inglês, *direct oral anticoagulants*; AVK = antagonistas da vitamina K; TEV = tromboembolismo venoso; EP = embolia pulmonar; TVP = trombose venosa profunda. Adaptada de American Society of Hematology 2020 guidelines^11^ .

A orientação não serve para todos pacientes, como no caso dos portadores de SAF triplo positivo – quando os DOACs são contraindicados.

Não se sugere um DOAC sobre outro.

Recomenda-se, com alto nível de evidência, o tratamento inicial com HBPM subcutânea (SC), HNF endovenosa (EV) ou SC monitorizada, dose fixa de HNF SC, ou fondaparinux SC, seguida de anticoagulantes orais a longo prazo.

Recomenda-se a duração do tratamento inicial (HNF, HBPM ou fondaparinux) por pelo menos 5 dias, associado aos AVKs desde o 1º dia de tratamento, até que o RNI (relação de normatização internacional) esteja no nível terapêutico, entre dois e três, por 2 dias consecutivos. A partir desse momento, as drogas parenterais podem ser suspensas com segurança. Não recomendamos, em hipótese alguma, o tratamento inicial para TVP somente com AVKs, uma vez que está associado a altas taxas de recorrência sintomática e risco de necrose cutânea. Uma alternativa a AVK é o uso de DOACs, como a edoxabana e a dabigatrana, a partir do 5º dia após heparinização ou apixabana ou rivaroxabana sem necessidade de ponte inicial com anticogulante parenteral.

#### HBPM

As HBPMs têm biodisponibilidade previsível, portanto, não se recomenda a monitorização dos níveis de antifator Xa como rotina^42,45^ (A). No entanto, devem ser realizados hemogramas com contagem de plaquetas. Podem ser administradas uma ou duas vezes ao dia, em regime hospitalar ou domiciliar.

#### HBPM versus varfarina

Tratamento a longo prazo (mais que 35 dias) com HBPM é tão efetivo quanto varfarina, para prevenção de mortes e recorrência de TEV, com risco similar de hemorragias.

#### HBPM versus HNF

Comparado com as HNFs, as HBPMs no tratamento prolongado (3 a 6 meses) apresentam risco menor de hemorragia grave e redução de recorrência de TVP proximal, com menos efeitos adversos. Em pacientes com EP aguda, as HBPMs são tão efetivas e seguras quanto as HNFs. Em pacientes com TVP aguda e insuficiência renal grave, sugere-se o tratamento com HNF ao invés de HBPM.

#### HNF EV

Caso a HNF seja o tratamento inicial de escolha, recomenda-se que, após o *bolus* EV (80 U/Kg ou 5.000 U), sua administração em infusão venosa contínua (inicialmente na dose de 18 U/Kg/h ou 1.300 U/h) com ajuste de dose para obter e manter um TTPa entre 1,5 e 2,5 acima do basal.

#### HNF SC

Se utilizadas doses fixas, recomenda-se uma dose inicial de 333 U/Kg seguida de 250 U/Kg duas vezes ao dia, realizando tratamento de acordo com o peso do paciente, com ajuste de dose para obter e manter um TTPa entre 1,5 e 2,5 acima do basal.

#### HBPM versus fondaparinux

O fondaparinux não é inferior à HBPM em taxas de recorrência de TEV, hemorragia grave ou morte.

#### AVKs

Os AVKs devem ser iniciados no 1º dia de terapia, associados à HNF, HBPM ou fondaparinux, exceto nos pacientes que tenham contraindicação ao seu uso. Tratamento com AVK requer mensuração frequente do RNI e monitorização das interações alimentares e com outras drogas.

#### Varfarina

Alcançar o RNI em nível terapêutico (entre dois e três) o mais breve possível é importante, uma vez que diminui a duração do tempo de uso de anticoagulantes parenterais e os custos. Embora a dose de 5 mg tenda a prevenir a anticoagulação excessiva, a dose inicial de 10 mg pode alcançar mais rapidamente o RNI terapêutico.

Em revisão sistemática que avaliou a eficácia de uma dose inicial de 10 mg de varfarina comparada com 5 mg em pacientes com TEV, não foram observadas diferenças entre elas em relação à recorrência de TEV, às hemorragias menores ou graves e à duração do período de internação. Não há vantagem na retirada gradual, comparada com a retirada abrupta da varfarina, em termos de evitar a recorrência de TEV.

#### Anticoagulantes orais diretos

Nos últimos anos, quatro DOACs – rivaroxabana, edoxabana, apixabana e dabigatrana – foram disponibilizados no mercado brasileiro, com algumas vantagens quando comparados aos AVK, como dose fixa, menos interações medicamentosas e alimentares, início de ação rápido e meia-vida curta, bem como ausência de necessidade de monitoramento regular^55,56^ .

#### Rivaroxabana

A rivaroxabana é uma molécula, com peso molecular de 436 g/mol^20^ .

Seu mecanismo de ação consiste na inibição direta do fator Xa^57^ . O fármaco apresenta rápida absorção por via oral, concentração plasmática em 2 a 4h e meia-vida de 6 a 7h^56,57^ . O seu metabolismo é hepático e seu uso deve ser evitado em indivíduos com doença hepática moderada a grave^56,58^ . Sua excreção é por via renal^10,13^ e sua dose deve ser ajustada em pacientes com insuficiência renal crônica (IRC), *clearance* de creatinina (ClCr) entre 15 e 50 mL/min, seu uso não é recomendado com ClCr <15 mL/min^13^ . A rivaroxabana possui interação medicamentosa com fármacos inibidores da glicoproteína P e a XYP3A4^59,60^ .

O estudo Einstein DVT demonstrou que a rivaroxabana é tão eficaz quanto o uso de HBPM e varfarina no tratamento da TVP em relação à taxa de recorrência (2,1% *vs* 3%; p < 0,001 para não inferioridade) com mesmo risco de complicações. Estudos como o Einstein EXTENSION e Einstein CHOICE demonstraram ainda emprego factível com baixo risco de sangramentos graves em uso mais prolongado, de até 12 meses. A dose habitual sem insuficiência renal é de 15 mg, por via oral, de 12 em 12 horas por 21 dias e, após, 20 mg em dose oral única diária pelo tempo previsto para tratamento^56,61,62^ . Não há necessidade de uso de droga parenteral inicial concomitante. Seu efeito anticoagulante pode ser revertido pelo andexanet alfa^57,61,62^ .

#### Edoxabana

A edoxabana é um anticoagulante oral cuja atividade se dá mediante inibição direta do fator Xa^63,64^ . Sua absorção ocorre, principalmente, no duodeno e no jejuno proximal^65-67^ , e pode ser administrada junto com a alimentação^68,69^ . Sua absorção ocorre em 1 a 2 horas, e sua meia-vida é de 5,8 a 10,7 horas^70-75^ . A dose diária é de 60 mg, em dose única diária, iniciada após 5 dias de tratamento com HNF ou HBPM^76,77^ . Seu uso é contraindicado em pacientes com ClCr <15 mL/min^78^ . A edoxabana tem potencial para o uso em crianças (considerando os estudos pré-clínicos de Fase I)^79,80^ .

No estudo Hokusai-VTE, observou-se que após o tratamento inicial com heparinas, a edoxabana foi não inferior à terapia padrão com heparina e varfarina^81^ . A edoxabana foi mais eficaz que a varfarina em pacientes com idade ≥ 75 anos e naqueles com múltiplas comorbidades^74,75^ . A edoxabana também se mostrou eficaz e segura no tratamento de TEV associada ao câncer^80,82^ . Sua dose deve ser reduzida para 30 mg, dose única diária, em pacientes com perda moderada da função renal (CrCl de 15-50 nL/min), peso menor ou igual a 60 kg ou que utilize inibidores da glicoproteína P.

#### Apixabana

A apixabana é um anticoagulante oral, inibidor direto do fator Xa. Foi aprovada para uso médico no Brasil em julho de 2011, para tratamento e prevenção secundária do TEV^83-85^ . Sua absorção se dá, principalmente, no duodeno e no jejuno proximal^86-88^ . As concentrações plasmáticas máximas ocorrem em 3 a 4h, com meia-vida de 6h^86,87,89-91^ . O acompanhamento laboratorial da sua atividade não se faz necessária^92,93^ . Para o tratamento na fase aguda do TEV, recomenda-se a dose de 10 mg, via oral, duas vezes ao dia por 5 dias, depois, 5 mg, duas vezes ao dia^91,94,95^ . Não há necessidade do uso concomitante de anticoagulante parenteral. Para a terapia estendida de TEV após 6 meses de terapia anticoagulante inicial, pode ser usada a dose de 2,5 mg, por via oral, duas vezes ao dia, a fim de reduzir a recorrência^87,96-98^ . Não há dados adequados sobre seu uso em mulheres grávidas e amamentação^99^ . A segurança e a eficácia ainda não foram estabelecidas em pacientes com idade <18 anos^87,98-100^ . Não se indica o uso em pacientes com insuficiência hepática grave^87^ . O ajuste da dose com base apenas na função renal não é necessário no tratamento do TEV, porém está contraindicada em pacientes com ClCr <15 mL/min^87,100^ .

#### Dabigatrana

O etexilato de dabigatrana mostra-se um inibidor reversível do sítio ativo da trombina. Prescinde de monitoramento laboratorial. Seu uso não é recomendado em pacientes com insuficiência renal com CrCl <30 mL/min. Como os outros DOACs, é contraindicado em pacientes com doença hepática avançada^101^ .

Os estudos RE-COVER, RE-COVER II, RE-MEDY e RE-SONATE observaram não inferioridade em eficácia no tratamento da EP (fatal ou não fatal), EP e/ou recidiva de TVP com dabigatrana, quando comparada à terapia padrão com varfarina após o uso inicial de 5 a 10 dias de anticoagulação parenteral. Apresenta concentração máxima plasmática 0,5 a 2h após a administração, com pico de concentração plasmática após 6h, e meia-vida de 11h. Seu efeito colateral mais importante é o sangramento. Pode ter sua ação revertida pelo idarucizumabe. Além disso, a dabigatrana pode ser removida da circulação por hemodiálise.

O uso concomitante com indutores da glicoproteína P deve ser evitado. Seu uso deve ser iniciado após, pelo menos, 5 dias de anticoagulação parenteral e na dose de 150 mg, por via oral, de 12 em 12 horas em todo período de tratamento^101,102^ .

### Recomendação 6

Pode-se recomendar preferencialmente o uso de DOACs ao invés de AVKs no tratamento inicial do TEV. Classe II nível B53-55.

#### TEV não complicado, não provocado em paciente hígido

Anticoagulação domiciliar, preferencialmente com DOACs, sem necessidade de internação hospitalar. Após o tratamento inicial de TVP ou EP não provocada, sugere-se tempo indefinido de anticoagulação (recomendação condicional, certeza moderada), conforme mostra a Tabela 3.

**Tabela 3:** Anticoagulação indefinida em comparação com a interrupção da anticoagulação em pacientes com TEV não provocado após tratamento inicial.

**Desfecho**	**Risco relativo (IC95%)**	**Risco com suspensão da anticoagulação**	**Diferença de risco com anticoagulação por tempo indeterminado**
Mortalidade	0,75 (0,49-1,13)	18 por 1.000	5 mortes a menos por 1.000
Embolia pulmonar	0,29 (0,15-0,056)	29 por 1.000	21 eventos de EP a menos por 1.000
Trombose venosa profunda	0,20 (0,12-0,34)	63 por 1.000	50 eventos de TVP a menos por 1.000
Sangramento maior	2,17 (1,40-3,35)	5 por 1.000	6 eventos de sangramento a mais por 1.000

TEV = tromboembolismo venoso; EP = embolia pulmonar; TVP = trombose venosa profunda. Adaptada de American Society of Hematology 2020 guidelines^11^ .

#### Observações

Não se aplica a pacientes que estão em alto risco de sangramento.

Para pacientes com TVP e/ou EP que continuarão com um DOAC para prevenção secundária, sugere-se o uso de DOAC em dose padrão ou DOAC em dose reduzida (rivaroxabana 10 mg/dia ou apixabana 2,5 mg, duas vezes ao dia).

Recomendação condicional, certeza moderada, Tabela 4.

**Tabela 4:** Dose mais baixa em comparação com DOAC em dose padrão em pacientes que continuam em anticoagulação indefinida.

**Desfecho**	**Risco relativo (IC95%)**	**Risco com dose padrão**	**Diferença de risco com dose reduzida de DOAC**
Mortalidade	0,68 (0,10-4,57)	6 por 1.000	5 eventos de mortes a menos por 1.000
Embolia pulmonar	1,25 (0,54-2,91)	5 por 1.000	21 eventos de EP a menos por 1.000
Trombose venosa profunda	0,75 (0,36-1,53)	9 por 1.000	50 eventos de TVP a menos por 1.000
Sangramento maior	0,97 (0,34-2,80)	4 por 1.000	6 eventos de sangramentos a mais por 1.000

DOAC **=** do inglês, *direct oral anticoagulants*; EP **=** embolia pulmonar; TVP **=** trombose venosa profunda. Adaptada de American Society of Hematology 2020 guidelines^11^ .

Para pacientes com TVP e/ou EP com doença cardiovascular estável, em uso de ácido acetilsalicílico (AAS), sugere-se sua suspensão, se possível, pelo tempo de duração da anticoagulação.

Recomendação condicional, certeza muito baixa, Tabela 5.

**Tabela 5:** Suspensão do AAS em comparação com sua continuidade (AAS + anticoagulação).

**Desfecho**	**Risco relativo (IC95%)**	**Risco com suspensão da anticoagulação**	**Risco com antinuidade de AAS associado à anticoagulação**
Sangramento maior	1,26 (0,34-2,80)	29 por 1.000	6 sangramentos a mais por 1.000 (2 a menos por 21 a mais)

AAS **=** ácido acetilsalicílico. Adaptada de American Society of Hematology 2020 guidelines^11^ .

Observação: não se aplica a pacientes com recente evento coronário ou intervenção coronária.

#### Duração do tratamento

Para tratamento inicial do TEV, sugere-se anticoagulação por 3 a 6 meses contra anticoagulação a longo prazo (6 a 12 meses).

Recomendação condicional, certeza moderada, Tabela 6.

**Tabela 6:** Anticoagulação a longo prazo comparada com a de curto prazo para pacientes com TEV provocado por fator de risco transitório.

**Desfecho**	**Risco relativo (IC95%)**	**Risco com anticoagulação a curto prazo**	**Diferença de risco com anticoagulação a longo prazo**
Mortalidade	1,38 (0,85-2,23)	18 por 1.000	7 mortes a menos por 1.000
Embolia pulmonar	0,66 (0,29-1,151)	50 por 1.000	17 eventos de EP a menos por 1.000
Trombose venosa profunda	0,50 (0,29-0,95)	117 por 1.000	50 eventos de TVP a menos por 1.000
Sangramento maior	1,46 (0,78-2,73)	13 por 1.000	6 sangramentos a mais por 1.000

TEV = tromboembolismo venoso; EP = embolia pulmonar; TVP = trombose venosa profunda. Adaptada de American Society of Hematology 2020 guidelines^11^ .

Observação: para TEV provocado por fator de risco transitório, prevenção secundária não precisa ser considerada.

#### TEV provocado

Para pacientes que desenvolvem TEV provocado por fator de risco transitório e sem história de antecedente de TEV provocado, sugere-se a interrupção da anticoagulação após a conclusão do tratamento inicial.

Recomendação condicional, certeza moderada, Tabela 7.

**Tabela 7:** Anticoagulação a longo prazo comparada com a de curto prazo para pacientes com TEV provocado recorrente.

**Desfecho**	**Risco relativo (IC95%)**	**Risco com anticoagulação de curto prazo**	**Diferença de risco com anticoagulação a longo prazo**
Mortalidade	0,75 (0,49-1,13)	18 por 1.000	7 mortes a menos por 1.000
Embolia pulmonar	0,29 (0,15-0,056)	29 por 1.000	17 eventos de EP a menos por 1.000
Trombose venosa profunda	0,20 (0,12-0,34)	117 por 1.000	50 eventos de TVP a menos por 1.000
Sangramento maior	2,17 (1,40-3,35)	5 por 1.000	6 sangramentos a mais por 1.000 (3 a menos por 12 a mais)

TEV = tromboembolismo venoso; EP = embolia pulmonar; TVP = trombose venosa profunda. Adaptada de American Society of Hematology 2020 guidelines^11^ .

Para pacientes com EP com ecocardiografia e/ou biomarcadores compatíveis com disfunção ventricular direita, mas sem comprometimento hemodinâmico (EP submaciça), o painel sugere anticoagulação isolada em vez do uso rotineiro de trombólise em adição à anticoagulação

Recomendação condicional, baixa certeza.

Observação: a trombólise é razoavelmente considerada para os mais jovens com EP submaciça com baixo risco de sangramento.

Para pacientes com TEV que continuarão recebendo prevenção secundária, sugere-se o uso de anticoagulação sobre AAS.

Recomendação condicional, certeza moderada, Tabela 8.

**Tabela 8:** AAS comparado com anticoagulação para pacientes recebendo prevenção secundária para TEV prévio.

**Desfecho**	**Risco relativo (IC95%)**	**Risco com anticoagulação**	**Diferença de risco com aspirina**
Mortalidade	0,86 (0,31-2,35)	7 por 1.000	1 mortes a menos por 1.000 (5 a menos por 10 a mais)
Embolia pulmonar	3,10 (1,24-7,73)	5 por 1.000	11 eventos de EP a mais por 1.000
Trombose venosa profunda	3,15 (1,50-6,63)	8 por 1.000	17 eventos de TVP a mais por 1.000
Sangramento maior	0,49 (0,12-1,95)	5 por 1.000	3 eventos de sangramentos a menos por 1.000

AAS = ácido acetilsalicílico; TEV = tromboembolismo venoso; EP = embolia pulmonar; TVP = trombose venosa profunda. Adaptada de American Society of Hematology 2020 guidelines^11^ .

### Recomendação 7

Para o tratamento inicial do TEV associado à um fator de risco transitório maior e se histórico de TEV recorrente, é recomendada a anticoagulação por um período de 3 a 6 meses, em comparação com um tratamento a longo prazo, que se estende de 6 a 12 meses. Classe II nível B^11,53,54^ .

### Recomendação 8

Em pacientes apresentando TEV recorrente associado a fator de risco persistente é recomendada a anticoagulação oral por tempo indeterminado. Classe I, nível B^103^ .

### Recomendação 9

Em pacientes em seu primeiro episódio de TEV, fator de risco não identificável ou persistente e baixo risco de sangramento deve ser considerada a profilaxia secundária com anticoagulantes. Classe IIa, nível A^103-111^ .

### Recomendação 10

Após o tratamento inicial de TEV não provocada, é recomendado um regime indefinido de anticoagulação. Para pacientes que permanecerão com DOAC para prevenção secundária, a sugestão é a utilização do DOAC em dose padrão ou em dose reduzida (por exemplo, rivaroxabana 10 mg/dia ou apixabana 2,5 mg, duas vezes ao dia). Classe II nível B^11,53,54^ .

### Recomendação 11

Em pacientes com indicação de profilaxia secundária do TEV não associado a câncer, recomenda-se o uso de DOACs em baixas doses (rivaroxabana 10 mg/dia ou apixabana 2,5 mg de 12 em 12 h) sobre DOACs em doses maiores, dicumarínicos ou AAS. Classe IIa, nível A^110,111^ .

### Recomendação 12

Em pacientes que estão sob profilaxia secundária devido a TEV, recomenda-se o uso de anticaoagulação sobre uso de AAS. Classe II nível B^11,53,54^ .

### Recomendação 13

Em pacientes com indicação de profilaxia secundária do TEV e SAF, recomenda-se a profilaxia secundária com o uso de dicumarínicos. Classe I, nível A^112,113^ .

### Anticoagulação no TEV agudo na IRC

A IRC é uma doença de caráter paradoxal no tratamento do TEV, pois, ao mesmo tempo que aumenta o risco de recorrência devido à disfunção endotelial, hiperreatividade plaquetária inicial, aumento da formação de fibrina e diminuição da atividade fibrinolítica, também aumenta o risco de hemorragia maior, com a piora progressiva da doença, devido à diminuição da agregação plaquetária^114^ .

Esse equilíbrio tênue entre o risco de recorrência e de hemorragia torna o tratamento do TEV um desafio especialmente naqueles com IRC de moderada a grave (ClCr entre 15 e 59 mL/min) ou IRC terminal (ClCr < 15 mL/min)^114^ .

Pacientes com IRC e TEV são tradicionalmente tratados com HNF, por via endovenosa, seguido de varfarina, com RNI alvo entre 2 e 3^115^ . Para pacientes com TEV e IRC terminal (CrCl <15 mL/min), as HBPM, a fondaparinux e os DOACs não são recomendados^115,116^ . Já na IRC de moderada a grave (CrCl entre 15 e 59 mL/min), as HBPM ou fondaparinux podem ser prescritos com cautela, em dose ajustada, eventualmente, com monitoramento dos níveis de anti-Xa, ou não recomendado, para evitar a bioacumulação e sangramento, particularmente na IRC avançada (CrCl <30 mL/min)^115,117^ .

A eficácia e segurança de DOACs, com eventuais ajustes de dose, em pacientes com TEV e com IRC leve a moderada (CrCl entre 30 e 80 mL/min) foram confirmadas no tratamento inicial e de extensão do TEV^115^ . A edoxabana é o único dos DOACs que prevê a redução de dose no tratamento agudo do TEV para pacientes com IRC (ClCr entre 30 e 50 mL/min), de 60 para 30 mg, via oral, uma vez ao dia, mantendo a mesma eficácia e segurança^118^ .

Nesses pacientes, é fundamental a reavaliação periódica da função renal.

### Anticoagulação no TEV agudo em mulheres

Nos países desenvolvidos, onde as causas clássicas de óbito materno na gestação, como eclâmpsia e hemorragia, foram controladas, o TEV passou a ser a principal preocupação, e sua prevenção, embasada em diretrizes apoiadas por evidências, com a instituição de farmacoprofilaxia em pacientes selecionadas, é ainda a melhor estratégia para reduzir essa complicação, potencialmente fatal.

A escolha dos métodos contraceptivos e de terapia de reposição hormonal (TRH) devem ser feitas por criteriosa seleção, avaliando os critérios de elegibilidade, as contraindicações e a autonomia das pacientes. Tanto o seu uso indiscriminado quanto a proibição imotivada são condutas inadequadas. Os contraceptivos hormonais e a TRH aumentam o risco de TEV; porém, as mulheres não devem ser privadas dos seus benefícios, além da contracepção, que as tornam mais livres na idade fértil e menos sintomáticas no climatério.

A fertilização assistida e terapia hormonal (TH) em mulheres transgênero também estão associadas ao desenvolvimento de TEV, e sua prevenção vai depender da indicação correta e da adequada profilaxia naquelas pacientes consideradas de alto risco para o desenvolvimento dessa doença.

#### TEV no ciclo gravídico-puerperal

As gestantes apresentam os três componentes etiopatogênicos da tríade de Virchow: a) estase venosa, causada pela compressão da VCI e veia ilíaca comum esquerda pelo útero gravídico e pela redução do tônus venoso pela ação miorrelaxante da progesterona; b) hipercoagulabilidade, secundária à indução da síntese hepática dos fatores VII, VIII e X da coagulação pelo estriol placentário, aumento dos níveis de fibrinogênio e inibidores dos ativadores do plasminogênio I e II e redução da síntese da proteína S; c) lesão endotelial, que ocorre durante a nidação, remodelamento endovascular das artérias espirais uterinas e expulsão da placenta^119,120^ .

Durante a gravidez, o risco de TEV aumenta entre cinco e dez vezes, podendo ser 35 vezes maior no puerpério quando comparada com a taxa entre mulheres não grávidas da mesma idade. Após o parto, a sua prevalência diminui rapidamente; mas há um risco residual por até cerca de 12 semanas. Aproximadamente dois terços das TVP ocorrem durante o período gestacional, distribuídos igualmente pelos 3 trimestres. No entanto, 43 a 60% dos episódios de EP ocorrem durante as primeiras 6 semanas do puerpério^119,120^ .

Entre as gestantes, quando comparadas com as não gestantes, as TVPs no membro inferior esquerdo (90% versus 55%) e no segmento iliofemoral (72% versus 9%) são ainda mais predominantes. Isso se deve à compressão acentuada da veia cava e da veia ilíaca comum esquerda contra a quinta vértebra lombar, causada pelo útero gravídico em crescimento^119,120^ .

Os principais fatores de risco para TEV durante a gestação e puerpério são: sobrepeso; obesidade; idade igual ou superior a 35 anos; trombofilias hereditárias ou adquiridas; viagens de longa distância; imobilidade; internação hospitalar no período gestacional; multiparidade; tabagismo; comorbidades, como por exemplo doença inflamatória intestinal; infecção do trato urinário; lúpus sistêmico eritematoso; hipertensão arterial sistêmica induzida pela gravidez ou pré-eclâmpsia; cirurgia pré-natal não obstétrica; hemorragia; transfusão e hiperêmese gravídica; entre outros. A prevenção do TEV na gravidez, por meio da aplicação de diretrizes baseadas em evidências ou modelos de avaliação de risco e a implementação de profilaxia mecânica e/ou farmacológica, ainda é a melhor estratégia para reduzir a taxa desses eventos^119,120^ .

Peculiaridades do tratamento anticoagulante durante a gestação e puerpério. A administração de varfarina entre a 6ª e a 12ª semanas de gestação pode induzir embriopatia fetal (hipoplasia nasal e/ou pontilhado das epífises), anormalidades do sistema nervoso central (displasia da linha média dorsal com agenesia do corpo caloso, atrofia do cerebelo linha média, displasia da linha média ventral com atrofia óptica e amaurose e hemorragia) e sangramento fetal^121^ . No entanto, a varfarina é segura e pode ser prescrita durante todo o período de amamentação^121^ . Os DOACs são contraindicados na gestação, por atravessarem a barreira placentária, e na amamentação, por passarem para o leite materno^121^ . Portanto, o tratamento do TEV nesses períodos deve ser preferencialmente feito com HNF, ou, preferencialmente, HBPM, quando houver restrições ao uso dessas^122^ .

O uso de fondaprinux durante a gestação tem indicação restrita e com evidência de baixa qualidade - a recomendação do ACCP geralmente era de grau 2C, indicando um grau moderado de recomendação baseado em evidências de baixa qualidade ou consenso de opiniões de especialistas^54,84^ .

### Recomendação 14

Durante a gravidez e amamentação, recomendamos contra o uso de DOACs Para tratamento de TEV na gestação, recomenda-se HNF ou HBPM. Classe I nível B (HNF x HBPM) e Classe I nível A (HBPM x AVK)^54,84,121,122^

#### Escolha da via de parto em mulheres em anticoagulação

A escolha da via de parto é obstétrica e não há contraindicação contra a maturação cervical artificial ou indução do parto. O parto da gestante em vigência de anticoagulação deve ser programado para o período entre 37 e 40 semanas^123^ . A HBPM deve ser suspensa 12h antes do parto se administrada em dosagens profiláticas, ou 24h antes se administrada em dosagens intermediárias ou terapêuticas, permitindo a administração segura de raquianestesia ou da anestesia peridural^123,124^ .

Independentemente da farmacoprofilaxia e da via de parto escolhida, a gestante deve fazer uso de meia elástica antitrombo durante todo o procedimento^4^ . Embora o risco associado à cesariana isoladamente seja baixo, a incidência de TEV aumenta quando existem outros fatores de risco associados. Portanto, a tromboprofilaxia deve ser prescrita, com base na estratificação de risco de cada gestante, com modelo de avaliação de risco validado^123,124^ .

#### TEV e contracepção

No Brasil, uma em cada cinco mulheres usa anticoncepcionais orais (ACOs) que oferecem benefícios que vão além da contracepção, como redução do sangramento menstrual, dismenorreia, síndrome pré-menstrual, enxaqueca, acne e hirsutismo. Além disto, os ACOs têm benefícios a longo prazo, como, por exemplo, a diminuição da incidência de câncer de endométrio, ovário e colorretal^122^ .

Os ACOs aumentam o risco de TEV de uma taxa inicial de 5/10.000 mulheres-ano entre não usuárias para 9-10/10.000 mulheres/ano entre usuárias. Para manter esse risco em perspectiva, é importante lembrar que o risco de TEV é de 29/10.000 durante o período gestacional e 300-400/10.000 no puerpério^122^ .

O risco tromboembólico dos ACOs está relacionado à dosagem de estrogênio e do tipo de progestágeno combinado a ele. ACOs antigos com altos níveis de estrogênio (>50 μg de etinilestradiol) estão associados a um risco maior de TEV do que os ACOs mais modernos (<50 μg de etinilestradiol). Não obstante, nenhuma redução de risco foi confirmada com os ACOs contendo 20 μg de etinilestradiol em comparação com aqueles que contém 30 μg de etinilestradiol^122^ . O tipo de progestágeno também influencia o risco de TEV, e os progestágenos de segunda geração (levonorgestrel [LNG] e noretisterona) são mais seguros do que os de terceira e quarta geração^122^ .

O risco de TEV associado a ACOs está vinculado ao aumento do peso corporal e da idade e com a reintrodução ou mudança de ACOs após suspensão superior a 4 semanas.

Entre as usuárias de ACOs, portadoras de trombofilias hereditárias têm maior risco de desenvolvimento de TEV. No entanto, devido à sua baixa prevalência na população geral e ao alto custo dos exames laboratoriais diagnósticos, a investigação de trombofilias hereditárias de rotina não é recomendada^122^ . A história pessoal ou familiar de TEV é um fator de risco mais importante e mais comum para TEV associado a ACOs^122^ . Os contraceptivos não orais, incluindo adesivos e anéis vaginais, também estão associados ao aumento do risco de TEV, elevando o seu risco em 7,9 e 6,5 vezes, respectivamente^122^ . A coagulação não apresenta alterações significativas com o uso de ACOs exclusivos de progesterona, implantes contendo LNG, sistema intrauterino de LNG ou injeção de medroxiprogesterona em depósito. Portanto, o seu uso é seguro nessa situação^122^ .

#### TEV e fertilização assistida

A fertilização *in vitro* (FIV) é a técnica mais utilizada para a reprodução humana em casais inférteis, e o TEV é uma complicação rara dessa técnica, ocorrendo em 0,1 a 2,4% dos ciclos de fertilização^10^ 2. O risco de TEV dobra nas gestações após uma FIV, quando comparado com o risco basal de gestantes não submetidas a ela. Isso se deve, particularmente, ao aumento de 5 a 10 vezes do risco durante o 1º trimestre de gestações após a FIV, secundário à síndrome de hiperestimulação ovariana (SHO), uma complicação iatrogênica e potencialmente fatal que ocorre em 33% de todos os ciclos gerados pela FIV. Mulheres que desenvolvem a SHO têm risco 100 vezes maior de TEV e, na SHO grave, a tromboprofilaxia com HBPM reduz a incidência do TEV, sem provocar aumento significativo de sangramento^122^ .

O TEV associado à FIV ocorre com maior frequência entre o 40º e 42º dia após a transferência do embrião, e tem a propensão de localizar-se em extremidades superiores e na região cervical em vez de no membro inferior esquerdo, local mais frequente nas gestações espontâneas^122^ . A FIV também aumenta o risco de trombose arterial, que ocorre em média no 10º dia após a transferência do embrião^122^ .

#### TEV e TRH

Embora dados recentes mostrem que os riscos da TRH podem superar os seus benefícios, muitas mulheres ainda são submetidas à TRH com estrógenos, de forma indiscriminada, para minimizar os sintomas do climatério. Essa conduta está associada ao aumento da incidência do TEV, principalmente no 1º ano de seu uso. Além disso, as mulheres com útero também recebem progestágenos para neutralizar o risco de câncer de endométrio na TRH, o que pode ser um fator de risco adicional para o desenvolvimento de TEV^122^ .

Estudos observacionais, revisões sistemáticas e metanálises relatam consistentemente um risco duas a três vezes maior de TEV entre mulheres pós-menopáusicas em TRH quando comparadas a mulheres pós-menopáusicas não submetidas à TRH^122^ .

O risco de TEV em mulheres submetidas à TRH é dependente da via de administração.

Os estrógenos por via oral provocam alterações pró-coagulantes, como aumento da resistência à proteína C ativada, por redução da concentração sérica da proteína S e redução da atividade fibrinolítica, provavelmente pela sua passagem e metabolização pelo fígado, porém, essas alterações não são observadas na TRH pela via trans. Não há dados adequados sobre seu uso em mulheres grávidas e amamentação dérmica^122^ .

Para prevenir TEV em mulheres que serão submetidas TRH, é importante identificar aquelas mais suscetíveis. Aquelas com história pessoal e familial de TEV são consideradas de alto risco e, portanto, não são candidatas à TRH com estrógenos por via oral^122^ .

A TRH, de fato, é o tratamento mais eficaz para minimizar os sintomas do climatério associados à queda dos níveis de estrogênio na menopausa. Porém, ela não deve ser prescrita de forma indiscriminada. Após avaliação dos seus riscos e benefícios, a melhor estratégia para proteger do risco de TEV é prescrição da TRH com a menor dose de estrógeno possível, por via transdérmica, isoladamente ou combinada com progestágenos micronizados, pelo menor tempo possível^123^ .

#### TEV em mulheres transgênero

Os termos transgênero e não conformidade de gênero descrevem uma situação em que a identidade de gênero de uma pessoa difere da anatomia sexual externa com a qual ela nasceu. Os objetivos da afirmação de gênero em mulheres transgênero são suprimir as características masculinas e induzir características femininas, na medida do possível.

A provisão de TH e da cirurgia para afirmação de gênero orientada por médico e amparada pelo sistema de saúde mostrou melhora na qualidade de vida e redução dos distúrbios observados nessa população, incluindo TEV^124^ .

Diversos estudos têm demonstrado um risco aumentado de TEV em mulheres transgênero que fazem uso de TH, e este risco está relacionado ao tipo e dosagem dos hormônios empregados e, principalmente, à sua via de administração. Sendo a administração transdérmica a via preferencial em mulheres transgênero com história pessoal ou familial de TEV ou naquelas portadoras de trombofilias hereditárias^124^ . Vale ressaltar que, para essa população, a TH não é eletiva, mas, sim, uma necessidade absoluta para se obter o fenótipo desejado, e em muitos lugares estão à margem da sociedade, não possuindo acesso aos profissionais ou serviços habilitados para prescrevê-la. Como consequência, muitas vezes a TH é obtida de forma ilegal e ingerida por conta própria, sem orientação profissional quanto à composição, doses e vias seguras. Outro ponto para reflexão é que as vias não orais da TH são usualmente mais caras do que a via oral e, portanto, inacessíveis para a maioria^124^ .

Uma estratégia plausível para atenuar o risco de TEV em grupos de risco é o início concomitante da anticoagulação profilática com a TH, especialmente nos primeiros 6 a 12 meses de tratamento^124^ . A prescrição adequada da contracepção e da TRH também demanda maturidade e conhecimento médico. A simples proibição do uso de ACOs e de TRH sem avaliar cuidadosamente os fatores de risco e a história familial e pessoal de cada paciente não interfere de forma decisiva na ocorrência de TEV e expõe desnecessariamente as mulheres ao risco de uma gestação indesejada e/ou a queda da qualidade de vida em qualquer faixa etária.

### Anticoagulação estendida – profilaxia secundária

A profilaxia secundária do TEV, também denominada “terapia estendida”, consiste na manutenção de terapia antitrombótica (anticoagulantes ou, menos frequentemente, antiagregantes plaquetários) após o período inicial de tratamento do TEV agudo (3 a 6 meses), com o intuito de prevenir sua recorrência.

A incidência de um novo evento de TEV após seu tratamento inicial pode superar os 40% em 10 anos^125^ . Porém, é possível identificar indivíduos com maior risco de recorrência e assim orientar a anticoagulação estendida para pacientes que irão se beneficiar de tal estratégia. Para a indicação da profilaxia secundária do TEV, deve-se levar em consideração dois fatores elementares na tomada de decisão: o risco de recorrência do TEV e o risco de sangramento do paciente sob anticoagulação. Dessa forma, o risco de recorrência pra TEV deve ser estimado, considerando que há fatores que caracterizam risco baixo, intermediário e alto, conforme pormenorizados na Tabela 1. Pacientes com baixo risco de recorrência não têm indicação de extensão profilaxia secundária^104,126^ . De maneira oposta, paciente com TEV de repetição sem fatores de risco identificáveis ou com fatores de risco persistentes costumam se beneficiar da extensão^103^ . Por fim, portadores de risco intermediário para TEV usualmente se beneficiam da profilaxia secundária, desde que apresentem baixo risco de sangramento^105-107^ .

Quanto maior o fator de risco transitório, menor a chance de recorrência, pois o TEV só se deu na presença de um estímulo trombótico forte. Já a ocorrência de TEV na presença de fatores de risco menores está associada a maior risco de recorrência. O racional dessa evidência consiste no fato de que pacientes que desenvolveram TEV mesmo na presença de estímulos fracos apresentam maior propensão à recorrência^105^ , conforme apresentado na Tabela 9.

**Tabela 9:** Risco de recorrência pós-TEV e risco de sangramento associado ao uso de anticoagulantes.

Risco de recorrência do TEV		Risco de sangramento associado ao uso de anticoagulantes
Risco	Exemplos	Idade > 75 anos, sangramento prévio sem causa tratável ou reversível, câncer ativo, AVE prévio, uso concomitantes de antiplaquetários ou AINH, insuficiência renal crônica, doença hepática crônica, pacientes frágeis, controle precário da anticoagulação (para o uso de dicumarínicos).
Baixo (< 3% a.a.)	Fatores de risco transitórios maiores ou reversíveis: cirurgia de grande porte, politrauma, internação clínica.	
Intermediário (3-8% a.a.)	Cirurgias menores, doença inflamatória intestinal, doença autoimune, uso de estrógenos, gravidez/puerpério, trombose associada a viagens longas, trombofilias (exceto SAF), TEV sem fator de risco identificável.	
Alto (> 8% a.a.)	Câncer ativo, SAF, TEV recorrente sem fator de risco identificável.	

TEV = tromboembolismo venoso; AVE = acidente vascular encefálico; SAF = síndrome antifosfolipídeo. Adaptada de Konstantinides et al.^127^ .

A profilaxia secundária com AAS ou varfarina em baixas doses (meta de RNI entre 1,5-2,0) não demonstrou boa relação de eficácia e segurança na prevenção de recorrência TEV^128-130^ .

Mais recentemente, com o advento dos DOACs e a superioridade destes nos desfechos de segurança^108-111^ , novas investigações foram realizadas, validando seu uso no tratamento inicial e estendido do TEV. Tais estudos são apresentados na Tabela 10. Os DOACs têm demonstrado, em seus estudos de Fase IV, resultados de eficácia e segurança similares aos ensaios clínicos randomizados de Fase III^131-141^ .

**Tabela 10:** Comparativo entre resultados de desfechos de segurança entre os DOACs.

**Estudo**	**Grupos, N**	**Redução de recorrência TEV/morte com DOACs**	**Sangramento maior (%)**	**Sangramento maior + Clin, Relevante %**
RE-SONATE	Dabigatrana, 681	-92% (0,08; 0,02-0,25)*	Dabigatrana: 0,3	Dabigatrana: 5,3
	Placebo, 662		Placebo: 0,0	Placebo: 1,8*
EINSTEIN-EXT	Rivaroxabana, 602	-82% (0,18; 0,09-0,39)*	Rivaroxabana: 0,7	Rivaroxabana: 6,0
	Placebo, 595		Placebo: 0,0	Placebo: 1,2*
AMPLIFY-EXT	Apixabana 2,5, 840	-81% (0,33; 0,22-0,48; A 2,5 mg *versus* placebo)	Apixabana 2,5: 0,2	Apixabana 2,5: 3,2
	Apixabana 5,0, 813	-80% (0,36; 0,25-0,53; A 5 mg *versus* placebo)*	Apixabana 5,0: 0,1	Apixabana 5,0: 4,3
	Placebo, 829		Placebo: 0,5	Placebo: 2,7
RE-MEDY	Dabigatrana, 1.430	1,3% varfarina	Dabigatrana: 0,9	Dabigatrana: 5,6*
	Varfarina, 1.426	1,8% dabigatrana	Varfarina: 1,8	Varfarina: 10,2
		(1,44; 0,78-2,64)		
EINSTEIN CHOICE	Rivaroxabana 20 mg, 1.121	-66% (0,34; 0,20-0,59; Riva 20 mg *versus* AAS)*	Riva 20: 0,5	Riva 20: 3,3
	Rivaroxabana 10 mg, 1.136	-74% (0,26; 0,14-0,47; Riva 10 mg *versus* AAS)*	Riva 10: 0,4	Riva 10: 2,4
	AAS 100 mg, 1.139		AAS 100: 0,3	AAS 100: 2,0

DOAC = do inglês, *direct oral anticoagulants*; TEV = tromboembolismo venoso; AAS = ácido acetilsalicílico.

A dabigatrana mostrou superioridade ao placebo e não inferioridade à varfarina na redução da recorrência do TEV. Comparativamente ao placebo, a dabigatrana apresentou mais eventos de sangramento, embora sem diferença estatística nos eventos de sangramento maior. A dabigatrana apresentou superioridade na redução de eventos de sangramento clinicamente relevante e não inferioridade na incidência de sangramentos maiores que a varfarina^108^ .

A eficácia e segurança da rivaroxabana foram testadas em duas investigações distintas^1,28,110^ . Na primeira, a rivaroxabana em dose terapêutica (20 mg/dia) demonstrou superioridade em eficácia quando comparada ao placebo, porém com mais eventos de sangramento (sem diferença em sangramento maior). Um segundo estudo de três grupos comparou duas doses distintas de rivaroxabana (10 e 20 mg/dia) *versus* AAS 100 mg/dia. As duas doses de rivaroxabana apresentaram eficácia similar entre si na prevenção da recorrência de TEV, sendo ambas superiores ao AAS. Não houve diferença de sangramento entre os grupos.

Um estudo foi dedicado para determinar a eficácia e segurança da apixabana na redução da recorrência do TEV. Duas doses de apixabana (2,5 e 5,0 mg de 12 em 12 horas) foram comparados a placebo, ambas apresentando superioridade em eficácia e não inferioridade em segurança^111^ . É importante mencionar que pacientes com alto risco de sangramento eram sistematicamente excluídos dos estudos supracitados.

#### Dose e escolha do agente antitrombótico

Embora a varfarina e o AAS tenham demonstrado superioridade sobre o placebo na prevenção de recorrência do TEV, estudos posteriores demonstraram a superioridade dos DOACs para tal finalidade. Dessa forma, AVKs e AAS devem ser usados para tal propósito apenas em situações em que os DOACs estão contraindicados, como, por exemplo, na SAF^112^ . Em único estudo comparativo entre a varfarina e a rivaroxabana em portadores de SAF triplo positivo, a varfarina demonstrou superioridade no desfecho combinado de eficácia e segurança, sendo a droga de escolha para esse grupo de pacientes^113^ . No entanto, não está claro se todos os pacientes devem receber doses reduzidas. Em pacientes com alto risco de recorrência e baixo risco de sangramento, o uso de DOACs em dose plena parece ser a escolha adequada.

#### Duração do tratamento estendido do TEV

Não há uma definição rígida da duração ideal do tratamento estendido do TEV. Na maioria dos ensaios clínicos que investigaram tal estratégia, a duração foi de 1 a 2 anos, com poucos ultrapassando os 36 meses. Porém, para pacientes com TEV recorrente sem fatores de risco identificáveis ou persistentes, recomenda-se a anticoagulação por tempo indeterminado^103^ . O termo “anticoagulação perene” deve ser evitado, uma vez que a manutenção da terapia anticoagulante deve ser periodicamente reavaliada, considerando o equilíbrio entre o risco para recorrência do TEV e o risco de sangramentos.

### Tratamento intervencionista no TEV agudo

#### Filtro de VCI

O uso de filtros de VCI tem sido associado a inúmeras controvérsias nos últimos anos. Há relatos de uso excessivo para indicações questionáveis e falhas na recuperação dos mesmos, que foram projetados para serem temporários, com subsequentes complicações trombóticas. Deve-se notar que o único objetivo dos filtros VCI é prevenir a EP e, portanto, reduzir a sua morbimortalidade. No entanto, os filtros VCI são a única opção de tratamento viável para pacientes TVP onde a anticoagulação é contraindicada, embora estudos randomizados sejam necessários.

No estudo Prevention du Risque d'Embolie Pulmonaire par Interruption Cave (PREPIC)^142^ , 400 pacientes com TVP proximal, com ou sem EP sintomático concomitante, foram randomizados para filtro de VCI permanente ou sem filtro. O estudo empregou um fatorial dois a dois, e os pacientes também foram randomizados para HBPM SC (enoxaparina) ou HNF IV (visando a uma relação TTPA de 1,5 e 2,5). Todos os pacientes foram submetidos a varreduras de perfusão/ventilação inicial e de acompanhamento quando os sintomas de potencial EP ocorreram, ou entre 8 e 12 dias para avaliar a EP assintomática. O desfecho primário foi a ocorrência de EP (sintomática ou assintomática) dentro de 12 dias após a randomização. Uma variedade de resultados secundários baseados em sintomas também foi avaliada. No dia 12, EP sintomática ou assintomática ocorreu em apenas dois pacientes (1,1%) no grupo de filtro versus nove pacientes (4,8%) no grupo sem filtro. Até 2 anos, houve seis eventos de EP no grupo com filtro (um óbito) e 12 no grupo sem filtro (cinco óbitos; OR 0,5; IC95% 0,19-1,33, p=0,16), mas TVP recorrente ocorreu em 37 pacientes (20,8%) no grupo de filtro versus 21 pacientes (11,6%) designados para nenhum filtro (OR 1,87; IC95% 1,1-3,2; p=0,02). As taxas de mortalidade foram semelhantes nos dois grupos em 2 anos (43 pacientes *versus* 40 pacientes nos grupos com filtro e sem filtro, respectivamente). Os resultados de 8 anos foram publicados em 2005 e mostraram que a EP sintomática ocorreu em nove pacientes (6%) no grupo com filtro versus 24 (15%) no grupo sem filtro (p = 0,008). No entanto, TVP recorrente foi mais comum no grupo com filtro (57 pacientes *versus* 41 pacientes; p=0,042). Os autores concluíram que, apesar da redução do risco de EP, o aumento do risco de TVP recorrente e a falta de benefício na sobrevida significam que o uso sistemático de filtros IVC não pode ser recomendado para essa população.

No estudo PREPIC 2^144^, publicado em 2015, randomizado e prospectivo, foram analisados 399 pacientes com EP e TVP sintomáticas. O grupo de implante de filtro de VCI removível foi constituído por pacientes que apresentavam pelo menos um critério de gravidade, isso é, maiores de 75 anos, câncer ativo, insuficiência cardíaca ou respiratória crônica, acidente vascular cerebral isquêmico com paralisia da perna nos últimos 6 meses (mais de 3 dias antes da randomização), TVP envolvendo o segmento iliocava ou bilateral, ou pelo menos um sinal de disfunção ventricular direita ou lesão miocárdica (n = 200) e grupo apenas anticoagulação (n = 199). Os dois grupos, com e sem filtro, foram anticoagulados por 6 meses, e se planejou a remoção do filtro em 3 meses. O filtro foi inserido com sucesso em 193 pacientes, e foi recuperado conforme planejado em 153 dos 164 pacientes nos quais a recuperação foi tentada. Aos 3 meses, EP recorrente ocorreu em seis pacientes (3,0%; todos fatais) no grupo do filtro, e em três pacientes (1,5%; 2 fatais) no grupo controle (risco relativo com filtro, 2,00 [IC95% 0,51-7,89]; p = 0,50). Os resultados foram semelhantes aos 6 meses. Nenhuma diferença foi observada entre os dois grupos em relação aos outros resultados. A trombose do filtro ocorreu em três pacientes. Conclui-se que entre os pacientes hospitalizados com EP aguda grave, o uso de um filtro de VCI removível associado à anticoagulação, em comparação com a anticoagulação isolada, não reduziu o risco de EP sintomática recorrente em 3 meses. Esses achados não suportam o uso desse tipo de filtro em pacientes que podem ser tratados com anticoagulação.

No estudo Filter Implantation to Lower Thromboembolic Risk in Percutaneous Endovenous Intervention (FILTER-PEVI)145, 141 pacientes submetidos à remoção precoce de trombos foram randomizados para filtro VCI ou sem filtro. Pacientes com e sem EP na apresentação foram incluídos. Apenas pacientes sintomáticos foram investigados e EP foram identificados em um paciente no grupo de filtro, em comparação com oito no grupo sem filtro. No entanto, não houve diferença na mortalidade e o estudo foi enfraquecido pela falta de imagens pré-operatórias das artérias pulmonares.

A contraindicação absoluta para anticoagulação inclui sangramento ativo incontrolável, alto risco de sangramento maior (por exemplo, defeito de coagulação, trombocitopenia grave, hemorragia intracerebral recente ou lesão cerebral com alto risco de sangramento) ou cirurgia urgente que exija interrupção da anticoagulação^143^ .

Parece haver consenso com evidência de boa qualidade para que a anticoagulação não seja usada concomitantemente ao implante do filtro de VCI, assim como em casos de contraindicação temporária à anticoagulação, em pacientes com filtro já implantado, este seja reiniciada tão logo o risco de sangramento esteja resolvido^11,54,84,144^ .

### Recomendação 15

Em pacientes com quadro agudo de EP, sob uso de anticoagulação, recomenda-se contrariamente a inserção de filtro de VCI. Classe II nível B^143,145,146^

### Recomendação 16

Em pacientes com TVP proximal e contraindicação formal ao início da anticoagulação, ou até os 03 meses iniciais de tratamento, recomendamos inserção de filtro temporário de VCI. Classe II nível B^143,145,146^

### Recomendação 17

Em pacientes com EP aguda e um filtro de VCI inserido como alternativa à anticoagulação, recomendamos terapia anticoagulante convencional se o risco de sangramento for resolvido. Classe II nível B^143,145,146^

#### Tratamento intervencionista do TEV agudo – trombólise, trombectomia farmacomecanica

Existem muitos trabalhos publicados sobre o tratamento intervencionista do TEV agudo, mas somente quatro trabalhos prospectivos randomizados têm melhor peso para análise dos resultados: CaVenT, ATTRACT, ATTRACT (Fase III) e CAVA^147-153^ .

O estudo CaVenT comparou trombólise direta por cateter (TDC) adjuvante com rtPA, além do tratamento anticoagulante com tratamento anticoagulante isolado seguido por anticoagulantes orais continuados em dois grupos por pelo menos 6 meses. O resultado primário de eficácia foi a presença de SPT, definido como uma pontuação ≥ 5 na escala de Villalta no membro acometido ou úlcera nessa perna na consulta de 24 meses. O estudo demonstrou uma redução significativa no SPT no grupo de intervenção, com uma redução de risco absoluto de 14,4% em 24 meses^147-149^ . Os resultados de acompanhamento a longo prazo do estudo CaVenT demonstraram que a redução de risco absoluto aumentou para 28% após 5 anos. O número necessário para tratar diminuiu de sete para quatro^147^ , sendo que nenhuma diferença na qualidade de vida foi detectada, mas o estudo não considerava esse desfecho. Foi evidenciada uma piora significativa na qualidade de vida para pacientes que desenvolveram SPT em toda a população do estudo^148^ . Além disso, uma análise de custo-efetividade demonstrou um benefício líquido do tratamento, com um incremento na taxa de custo-efetividade de US$ 20.000 na qualidade de vida, ajustado por ano/vida^150^ .

O estudo ATTRACT é um estudo prospectivo randomizado multicêntrico para avaliar trombectomia farmacomecânica (TFM) e TDC para a prevenção de SPT em pacientes com TVP femoral ou mais proximal em comparação com a terapia padrão com anticoagulantes orais isoladamente^151^ . O protocolo usou três modalidades diferentes (TDC isolado ou combinado com TFM usando Angiojet®/Trellis-8^®^) nos pacientes randomizados para o grupo de tratamento, a critério do médico assistente. O resultado de eficácia primária foi semelhante ao do estudo CaVenT, ou seja, a presença de SPT, definida como uma pontuação ≥5 na escala de Villalta no membro acometido ou uma úlcera, ocorrendo a qualquer momento desde a visita de acompanhamento pós-randomização de 6 meses até a visita de 24 meses (inclusive). Ao longo de 24 meses, não houve diferença na proporção de pacientes que desenvolveram SPT entre os dois grupos de tratamento (47% com TDC/TFM versus 48% com terapia padrão; RR 0,96; IC95% 0,82-1,11; p = 0,56). TFM levou a mais sangramentos graves em 10 dias (1,7% TFM versus 0,3% terapia padrão; p = 0,049) e nenhuma diferença em TEV recorrente em 24 meses (12,5% TFM versus 8,5% terapia padrão; p = 0,087). A intervenção reduziu a dor e o edema nas pernas até 30 dias, mas não melhorou significativamente a qualidade de vida desde o início do tratamento até 24 meses. A intervenção reduziu significativamente os escores de gravidade de SPT e reduziu significativamente o desenvolvimento de SPT moderado a grave (18% com SPT versus 24% com terapia padrão; RR 0,73; IC95% 0,54-0,98; p = 0,035) ao longo de 24 meses de seguimento.

Já o estudo ATTRACT (Fase III)^152^ foi um ensaio clínico randomizado de Fase III, multicêntrico, aberto, cego para avaliadores. Foi utilizado o Angiojet^®^ para TFM, e, em casos em que o trombo se estendia mais distal à poplítea, poderia-se realizar previamente TDC, e posteriormente TFM. Evidenciou-se que na TVP poplíteo-femoral, exceto pela redução do SPT aos 6 meses de acompanhamento (21,7% com TFM *versus* 40,8% com controle; p=0,01), não foram identificadas diferenças significativas nos resultados iniciais ou tardios do estudo entre o TFM e do braço de controle dos pacientes. Na TVP fêmoro-ilíaca, a SPT foi em 41 (43,2%) de 95 pacientes tratados com TFM e 40 (40,0%) de 100 pacientes do braço de controle (p = 0,65) em 24 meses.

As estimativas da escala de Villalta e dos escores VCSS de 6 a 24 meses e das proporções de pacientes com SPT em 6 meses (26,2% *versus* 39,2%; p = 0,08) foram nominalmente menores para TFM do que para controle, mas as diferenças não foram estatisticamente significantes e nem foram observadas diferenças nas ocorrências de 2 anos de SPT moderada ou grave, SPT grave, ulceração venosa ou QOL de 6 a 24 meses.

A melhora na gravidade da doença inicial foi maior para a TFM do que para o controle, alcançando significância estatística em 30 dias (diferença média de 0,84 pontos na escala Likert; p = 0,0061). Da linha de base até 30 dias, os tratados por TFM tiveram melhora na qualidade de vida (diferença de 12,6 pontos da escala VEINES-QOL; p=0,0001) e sintomas (diferença de 11,4 pontos da escala VEINES-Sym; p=0,0003) do que os pacientes do braço de controle. Aos 30 dias, os tratados por TFM também apresentaram menos trombo residual. Em relação a complicações, sangramento maior, EP, insuficiência renal e bradicardia foram pouco frequentes com TFM (< 2% cada), mas a recorrência de TEV em 24 meses foi mais frequente (13,9% com TFM versus 6,8% com controle; p = 0,03).

O ensaio clínico CAVA é um estudo prospectivo, randomizado, multicêntrico, comparando o uso de trombólise acelerada por ultrassom versus apenas terapia de anticoagulação padrão^153^ . Em contraste com o ATTRACT, este estudo envolveu apenas TVP fêmoro-ilíaco, abordando assim uma das críticas levantadas sobre os critérios de inclusão do estudo ATTRACT. No geral, o CAVA randomizou 184 pacientes: 91 para intervenção e 93 para terapia padrão, com 77 recebendo terapia e 75 permanecendo no grupo de terapia padrão após falha na triagem/retirada do consentimento. Após 12 meses de acompanhamento, o estudo não mostrou diferença entre os dois grupos de tratamento, com 22 de 77 (29%) daqueles submetidos à intervenção versus 26 de 75 (35%) daqueles em terapia padrão desenvolvendo SPT (p = 0,42). Esses dados levaram à conclusão de que, para pacientes com TVP proximal aguda, a TFM não impediu a SPT, mas aumentou o sangramento maior. Os resultados sugeriram que pode haver um benefício na redução do risco de SPT moderada a grave, embora ainda houvesse uma taxa de SPT maior do que a esperada em pacientes tratados com remoção precoce do trombo^153^ . No entanto, nenhum benefício foi observado em pacientes com TVP poplítea-femoral^154^ .

Em uma revisão sistemática publicada na Cochrane^155^ , comparando estratégia trombolíticos *versus* anticoagulação para TVP de membro inferior, observou-se que aqueles que receberam trombólise tiveram mais complicações hemorrágicas (6,7% versus 2,2%) (RR 2,45; IC95% 1,58-3,78; 1.943 participantes, 19 estudos; evidência de qualidade moderada). Nenhuma diferença entre as estratégias foi detectada pela análise de subgrupo (p = 0,25). Até 5 anos após o tratamento, ocorreu um número ligeiramente menor de casos de SPT naqueles que receberam trombólise, 50% em comparação com 53% na anticoagulação padrão (RR 0,78; IC95% 0,66-0,93; 1.393 participantes, seis estudos; evidência de qualidade moderada). Isso ainda foi observado no acompanhamento tardio (além de 5 anos) em dois estudos (RR 0,56; IC95% 0,43-0,73; 211 participantes; evidência de qualidade moderada). Usando a análise de subgrupo para investigar se o nível de TVP (fêmoro-ilíaca, poplítea-femoral ou não especificado) teve efeito sobre a incidência de SPT, não foi observado nenhum benefício da trombólise independente do nível da TVP (seis estudos; teste para diferenças de subgrupo: p = 0,29). Trombólise sistêmica e TDC tiveram níveis semelhantes de eficácia. Estudos de TDC incluíram quatro ensaios em TVP femoral e fêmoro-ilíaca, e os resultados são consistentes com aqueles de ensaios de trombólise sistêmica em TVP em outros níveis de oclusão.

### Recomendação 18

Em pacientes selecionados com TVP aguda ilíaco-femoral sintomática (especialmente baixo risco de sangramento), recomendamos a utilização de estratégias precoces para remoção do trombo. Classe II nível B^147-153^

### Recomendação 19

Em pacientes com TVP comprometendo veias femorais, poplíteas e/ou de panturrilha, a remoção precoce do trombo não é recomendada. .Classe II nível C Referência^147-153^

### Recomendação 20

Em pacientes com TVP aguda no membro inferior que tenham sido submetidos à remoção do trombo, a duração da antiacoagulação deve seguir a mesma intensidade e duração que a indicada para pacientes sem o procedimento de remoção do trombo. Classe I nível B^147-153^ .

## COVID e TEV

### TEV relacionado à COVID-19

#### Epidemiologia

O risco aumentado de eventos tromboembólicos em pacientes com infecção pelo vírus SARS-CoV-2 tem sido demonstrado por diversos estudos^156-160^ , assim como maior mortalidade quando esses eventos estão associados, tornando necessária sua suspeição principalmente nos pacientes com infecção moderada a grave, internados em enfermarias ou em unidades de terapia intensiva.

Os eventos trombóticos no território venoso e suas consequências, que incluem as TVP e EP, são mais prevalentes em pacientes hospitalizados de uma forma geral, principalmente em unidades de terapia intensiva^161^ , sendo que a incidência de TEV em pacientes hospitalizados sem tromboprofilaxia é de 14,9%^161,162^ .

Nos pacientes com quadro de COVID-19 com necessidade de hospitalização, a TEV, segundo metanálise publicada no Chest Journal em 2021, foi de 17%, EP, 7,1% e TVP, 12,1%^163^ . Em outra recente metanálise, a prevalência de TVP e EP em pacientes hospitalizados com diagnóstico de COVID-19 foi ainda maior (14,8% e 16,5%, respectivamente)^164^ .

#### Diagnóstico

##### Exames laboratoriais

A dosagem de DD encontra-se aumentada na maioria dos pacientes com infecção pelo SARS-CoV-2 mesmo sem quadro de TEV, porém alguns estudos demonstraram níveis de DD ainda maiores nos pacientes com COVID 19 associada à TVP e EP^165-167^ .

Demelo-Rodríguez et al.^167^ relataram que um ponto de corte de dosagem de DD de 1,57 (μg/ml) possui sensibilidade de 95,7% e especificidade de 29,3%, valor preditivo positivo de 19% e valor preditivo negativo de 97,5% para diagnóstico de TVP assintomática nos pacientes com COVID-19. DD elevado também foi considerado como preditor de gravidade^168,169^ .

#### Tratamento

Os princípios do tratamento do TEV em pacientes com COVID-19 não diferem em relação aos pacientes com TEV não COVID^170^ . Recomenda-se uso de anticoagulantes parenterais como terapia inicial (HNF e HBPM), especialmente nos pacientes internados, podendo realizar a transição para anticoagulantes orais (DOACs ou AVKs) após estabilização clínica^171^ . Os DOACs têm sido a terapêutica preferencial na transição do regime parenteral para o regime oral, por apresentar menores taxas de sangramento do que a varfarina. Os inibidores do fator Xa, como a rivaroxabana e apixabana, têm sido os mais utilizados no tratamento do TEV em pacientes com COVID-19 após a alta hospitalar e estabilização do quadro clínico dos pacientes. A duração do tratamento dependerá da persistência ou não de fatores de risco, além de doenças concomitantes que necessitem de anticoagulação plena, porém deve ser considerada por no mínimo 3 meses^172^ .

#### Anticoagulação em pacientes com COVID-19 sem evidência de TEV

Esse assunto tem suscitado muitas dúvidas e estudos discrepantes na literatura. Não resta dúvida de que os pacientes com COVID-19 com evidências de TEV devem ser submetidos à anticoagulação em regime terapêutico habitual, o mesmo que seria administrada aos pacientes sem COVID-19. O cerne da questão é qual tipo de regime anticoagulante deve ser administrado nos pacientes com COVID-19 sem evidências de fenômenos trombóticos. Recentemente, duas publicações avaliaram os regimes de anticoagulação terapêutico e profilático em dois tipos de pacientes: os pacientes graves, submetidos à intubação orotraqueal, e os pacientes com COVID-19 moderado, internados, mas sem necessidade de oxigenioterapia suplementar. O primeiro estudo avaliou os tipos de anticoagulação nos pacientes graves, já submetidos à intubação orotraqueal, internados em UTI. Foram avaliados 1.098 pacientes: um grupo de pacientes submetido à terapia com HNF ou enoxaparina em dose terapêutica, e o outro grupo de pacientes submetido à enoxaparina ou HNF em dose profilática. Os resultados do estudo demonstraram que a sobrevida hospitalar nos pacientes em regime terapêutico foi de 62,7% *versus* 64,5% no grupo de pacientes submetidos ao regime profilático, sem diferença estatística entre os grupos. A taxa de hemorragia importante, entretanto, foi maior no grupo submetido à anticoagulação terapêutica no grupo profilático (3,8% *versus* 2,3%). Os autores concluíram que não houve diferença em relação à mortalidade nos pacientes graves submetidos aos tratamentos terapêutico ou profilático com anticoagulação, porém houve maior taxa de sangramento no grupo de pacientes submetidos à anticoagulação plena^173^ . O estudo INSPIRATION, que avaliou 600 pacientes internados em unidade de terapia intensiva (UTI), randomizados para tratamento com anticoagulação plena *versus* profilática, não encontrou benefícios do uso da anticoagulação terapêutica na prevenção de mortalidade, fenômenos tromboembólicos e uso de ECMO (HR 1,06; IC95% 0,83-1,36)^174^ .

Outro estudo teve como escopo a avaliação entre os regimes terapêuticos de anticoagulação plena *versus* profilático com enoxaparina ou HNF em pacientes internados com COVID-19 moderada, sem lesão ou insuficiência de órgão alvo e sem necessidade de oxigenioterapia suplementar. Entre 2.219 pacientes na análise final, a probabilidade de que a anticoagulação de dose terapêutica aumentou o suporte de órgão – livre em dias –, em comparação com a tromboprofilaxia de tratamento usual, foi de 98,6%. A probabilidade final da superioridade da dose terapêutica de anticoagulação em relação à tromboprofilaxia de tratamento usual foi de 97,3% em pacientes com DD alto, 92,9% em pacientes com DD baixo e 97,3% em pacientes com DD desconhecido.

Sangramento importante ocorreu em 1,9% dos pacientes que receberam dose terapêutica de anticoagulação, e em 0,9% dos que receberam tromboprofilaxia. Os autores concluíram que a anticoagulação plena com enoxaparina ou HNF em pacientes com COVID-19 moderada aumentou a probabilidade de sobrevida hospitalar, com menor incidência de insuficiência orgânica multisistêmica, ou seja, insuficiência renal, cardiovascular e respiratória^172,173^ . Da mesma forma, o estudo HEP-COVID avaliou 253 pacientes com COVID-19 e níveis elevados de DD, randomizando-os para doses terapêutica e profilática de anticoagulantes. Os desfechos primários (óbito, TEV e trombose arterial) foram maiores no grupo que recebeu anticoagulante profilático (42%) do que no grupo de pacientes que recebeu anticoagulação plena (29%) (RR 0,68; IC95% 0,49-0,96; p = 0,03)^175^ . Esse benefício só foi observado em pacientes que se encontravam em leito de enfermaria. Pacientes internados em UTI não foram beneficiados com a anticoagulação plena.

De maneira geral, com base na literatura, em pacientes críticos, internados em UTI com COVID-19, é recomendado o uso de anticoagulantes na dose profilática (enoxaparina 40 mg, SC, dose única diária). Já os pacientes internados com COVID-19 em regime de enfermaria, é recomendada a administração de doses terapêuticas de anticoagulantes (rivaroxabana 20 mg/dia ou enoxaparina 1 mg/kg/dose, SC, de 12 em 12 horas). Por sua vez, pacientes internados por outras condições clínicas, nos quais o COVID-19 é descoberto incidentalmente, a dose profilática de anticoagulante já é suficiente, sendo recomendada pela literatura em geral.

Um potencial benefício de se manter anticoagulante em doses profiláticas após a alta hospitalar de pacientes com COVID-19, com alto risco de TEV, foi demonstrado no estudo MICHELLE^176^ , o qual avaliou 320 pacientes com COVID-19 internados com alto risco de desenvolver TEV, mas sem nenhuma evidência de TEV documentada até o momento de alta, randomizados para receber rivaroxabana na dose de 10 mg e nenhum anticoagulante por 35 dias após a alta hospitalar. A incidência de complicações (TEV, tromboembolismo arterial ou evento cardiovascular) foi maior no grupo de pacientes que não recebeu anticoagulante (9%) do que no grupo que recebeu a dose profilática de rivaroxabana (3%) (RR 0,33; IC95% 0,12-0,90). Apesar desses dados robustos, essa é uma coorte pequena, portanto ainda não existe consenso na literatura acerca da recomendação de rotina de anticoagulantes em dose profilática para pacientes com alto risco de TEV que receberam alta hospitalar devido à COVID-19, sendo recomendado avaliar caso a caso, pesando o risco-benefício de cada paciente, enquanto se aguardam novos estudos na literatura.

Um outro estudo, que acompanhou ambulatorialmente 4.906 pacientes internados com COVID-19 que receberam alta hospitalar, evidenciou uma incidência de TEV de 1,6% pós-alta, sendo que farmacoprofilaxia para TEV só foi utilizada em 13% da amostra. A incidência de TEV foi menor nos pacientes que não receberam anticoagulantes do que nos pacientes submetidos a farmacoprofilaxia para TEV, na amostra desse estudo. A taxa de sangramentos maiores com uso de anticoagulantes foi de 1,7%, sendo maior que o risco de TEV^177^ .

A maior parte dos pacientes com COVID-19 são tratados ambulatorialmente, sem necessidade de internação hospitalar. Para esses pacientes, não é indicada farmacoprofilaxia para TEV, a menos que o paciente apresente outros fatores associados que necessitem o uso de anticoagulantes, como TEV já documentada atual, fibrilação atrial, câncer em atividade ou trombofilias hereditárias que exibam alto risco de TEV, tais quais deficiência da antitrombina, deficiência de proteína C, entre outras. Cada paciente deve ser avaliado individualmente, pesando-se o risco-benefício do uso de anticoagulantes como farmacoprofilaxia para TEV, nos pacientes com COVID-19, tratados ambulatorialmente, sem necessidade de internação.

Em resumo: o tratamento da TEV em pacientes com COVID-19 segue princípios semelhantes ao tratamento de TEV sem relação com o COVID-19. Inicialmente, recomenda-se o uso de anticoagulantes parenterais (HNF e HBPM) em pacientes hospitalizados, podendo transitar para anticoagulantes orais (DOACs ou AVKs) após a estabilização clínica. Os DOACs, como rivaroxabana e apixabana, têm sido preferenciais devido a menores taxas de sangramento em comparação com a varfarina.

Portanto, a decisão de aplicar a farmacoprofilaxia para TEV pós-alta deev ser personalizada, considerando o risco-benefício. Em pacientes ambulatorais, a profilaxia não é recomendada sistematicamente, exceto na presença de fatores de risco e devendo se levar em conta a chance de sangramento, assim como outras condições clínicas do paciente.

### Recomendação 21

Em eventos tromboembólicos agudos de pacientes infectados por COVID-19, recomendamos que a decisão da anticoagulação siga os mesmos princípios que os eventos que ocorram sem a concomitância da infecção viral. Classe II nível A^172-175^

### Recomendação 22

Em pacientes ambulatoriais infectados por COVID-19 e na ausência de outras condições clínicas que indiquem o uso de anticoagulantes, a administração desses medicamentos não é recomendada. Classe I nível B^172-175^

### Recomendação 23

Em pacientes de internação hospitalar pelo COVID-19, recomendamos categorizar o risco de evento tromboembólico venoso no pós-alta, antes de decidir pelo uso de anticoagulação ambulatorial. Classe 2 nível A^177,178^
